# Development and validation of resource-driven risk prediction models for incident chronic kidney disease in type 2 diabetes

**DOI:** 10.1038/s41598-021-93096-w

**Published:** 2021-07-01

**Authors:** Sarega Gurudas, Manjula Nugawela, A. Toby Prevost, Thirunavukkarasu Sathish, Rohini Mathur, J. M. Rafferty, Kevin Blighe, Ramachandran Rajalakshmi, Anjana R. Mohan, Jebarani Saravanan, Azeem Majeed, Viswanthan Mohan, David R. Owens, John Robson, Sobha Sivaprasad, the ORNATE India Study Group

**Affiliations:** 1 Institute of Ophthalmology, University College London, London, UK; 2 Nightingale-Saunders Clinical Trials and Epidemiology Unit, King’s College London, London, UK; 3 Department of Primary Care and Public Health, Imperial College London, London, UK; 4 Institute of Applied Data Science, Queen Mary’s University London, London, UK; 5 Swansea University, Wales, UK; 6 Madras Diabetes Research Foundation, Dr Mohan’s Diabetes Specialities Centre, Chennai, India; 7 School of Public, Health Imperial College London, London, UK; 8 NIHR Moorfields Biomedical Research Centre, University College London, City Road, London, 162EC1V 2PD UK

**Keywords:** Diagnosis, Disease prevention, Health services, Public health, Diseases, Endocrinology, Medical research, Nephrology, Risk factors

## Abstract

Prediction models for population-based screening need, for global usage, to be resource-driven, involving predictors that are affordably resourced. Here, we report the development and validation of three resource-driven risk models to identify people with type 2 diabetes (T2DM) at risk of stage 3 CKD defined by a decline in estimated glomerular filtration rate (eGFR) to below 60 mL/min/1.73m^2^. The observational study cohort used for model development consisted of data from a primary care dataset of 20,510 multi-ethnic individuals with T2DM from London, UK (2007–2018). Discrimination and calibration of the resulting prediction models developed using cox regression were assessed using the c-statistic and calibration slope, respectively. Models were internally validated using tenfold cross-validation and externally validated on 13,346 primary care individuals from Wales, UK. The simplest model was simplified into a risk score to enable implementation in community-based medicine. The derived full model included demographic, laboratory parameters, medication-use, cardiovascular disease history (CVD) and sight threatening retinopathy status (STDR). Two less resource-intense models were developed by excluding CVD and STDR in the second model and HbA1c and HDL in the third model. All three 5-year risk models had good internal discrimination and calibration (optimism adjusted C-statistics were each 0.85 and calibration slopes 0.999–1.002). In Wales, models achieved excellent discrimination(c-statistics ranged 0.82–0.83). Calibration slopes at 5-years suggested models over-predicted risks, however were successfully updated to accommodate reduced incidence of stage 3 CKD in Wales, which improved their alignment with the observed rates in Wales (E/O ratios near to 1). The risk score demonstrated similar model performance compared to direct evaluation of the cox model. These resource-driven risk prediction models may enable universal screening for Stage 3 CKD to enable targeted early optimisation of risk factors for CKD.

## Introduction

The prevalence of type 2 diabetes (T2D) and its complications are increasing rapidly around the world and chronic kidney disease (CKD) is one of the major complications^1,2^. In 2017, approximately 697 million people were reported to have reduced glomerular function worldwide, with 125 million being related to T2D^3^. The Kidney Disease: Improving Global Outcomes (KDIGO) group have developed clinical practice guidelines for improving the diagnosis and treatment of patients with diabetes and CKD based on estimated glomerular filtration rate (eGFR) and albuminuria^4^. Given the wide variability in the rate of decline of glomerular function in people with T2D, prediction of a decrease of eGFR from normal ranges to less than 60 mL/min/1.73m^2^ is especially challenging^5^.The kidney failure risk equation is useful for predicting more severe disease in people with CKD^6,7^. Early identification of high-risk group will allow more efficient use of available resources to implement prevention strategies to avoid or slow the rate of downward trajectory to end stage renal disease^8^.

Moreover, CKD shares common risk factors with diabetic retinopathy (DR) and cardiovascular disease (CVD) and identifying these complications incur significant healthcare costs^9–14^. Many low- and middle-income countries (LMIC) have limited laboratory facilities and clinical expertise to screen for these complications, where average spending on healthcare can range US$100-US$400 per capita per annum compared with US$2000 in high income countries^15,16^. Therefore, prediction models that identify early CKD in T2D have multiple beneficial effects in preventing multiple morbidities. However, there is a paucity of resource-friendly CKD risk models containing predictors that do not require costly technical or laboratory expertise and resources (table S1) that can be easily applied in LMICs^17,18^. In this manuscript we refer to the development of such predictor models as resource-driven models.

In this study, we considered these limiting factors and aimed to build resource-driven stage 3 CKD predictive models that could be applied globally and in resource-constrained environments.

## Methods

The Moorfields Research Management Committee approved the use of these fully anonymised UK retrospective datasets for model development and validation (SIVS1057). Approval was also obtained from the Caldecott guardian of these anonymised datasets in Queen Mary University London (QMUL) and Secure Anonymised Information Linkage (SAIL) in Wales. This study was conducted in accordance with the Declaration of Helsinki. Patient-level consent was not required as the study only used fully anonymised routinely collected data (SIVS1057, Moorfields Eye Hospital dated 14/04/2020).

### Study cohorts

We used two observational study cohorts derived from primary care electronic health record data for development and validation. The development cohort was extracted from a fully anonymised primary care dataset consisting of 105,533 people with T2D of multi-ethnic origin registered with 134 general practices (GP) in inner London(London cohort)^19^. Fully anonymised data from the SAIL databank^20,21^ was used for external validation, consisting of 140,157 T2D participants registered with over 170 primary care-GP practices from Wales of predominately White ethnicity (Wales cohort). The T2D and coding standards of both cohorts were consistent with the Quality outcomes Framework (QOF)^22^.

Both study cohorts enabled entry of individuals into the cohort at any time during the 11-year period (2007–2017). Cohort entry date was defined as the latest of date of 18th birthday, study start (01/01/2007) or date of registration with the GP. Follow-up time ended at the earliest date of study end (31/12/2017), de-registration from the GP, death or onset of CKD. We defined study baseline as the first T2D read code (a hierarchical dictionary of medical nomenclature)^23^ between entry and exit dates. Participants with a code for stage 3 + CKD, code for dialysis or eGFR < 60 ml/min/1.73 m^2^ on or prior to baseline were excluded from the cohort.

### Outcomes

The endpoint was defined as the latest of the first two eGFR readings below 60 mL/min/1.73m^2^ taken at least 3 months apart. Participants were also identified through read codes for dialysis or stage 3 CKD onwards^19^. Data collection extended a maximum of 11 years, but as baseline risk factor data may become less predictive with time, the study outcome was censored at 5 years. The eGFR in both datasets was measured using the 4-variable Modification of Diet in Renal Disease Study Eq. ^24^.

### Candidate predictors

Candidate predictors were pre-identified from review of literature (Table S1) prioritising variables that are commonly available in clinical practice and key confounders of CKD (age, sex and ethnicity). These variables included age at baseline, gender, duration of diabetes, ethnicity, HbA1c level, total cholesterol, HDL cholesterol, body mass index (BMI), albumin-creatinine ratio (ACR), eGFR, use of anti-hypertensives (anti-HTs), use of insulin, presence of CVD and sight threatening diabetic retinopathy (STDR).

### Sample size

The development and cohort consisted of 1,378 events allowing for a maximum of 68 parameters within guidelines for the 14 covariates considered, plus additional parameters for interactions, fractional polynomials or categorical variables^25–27^. The external validation cohort had adequate sample size achieving 10 events per parameter (656 events).

### Development of models

#### Full model (model 1)

We developed 5-year Cox proportional hazards models for predicting incident eGFR < 60 mL/min/1.73m^2^ using Stata version 16^28^. We used fractional polynomial terms to model non-linear relationships for the continuous variables where appropriate. All continuous variables were scaled and centred. eGFR was categorised (every 10 ml/min/1.73m^2^ interval) due to the recording of normal eGFR in electronic health record (EHR) data as eGFR > 90 mL/min/1.73m^2^. Albumin to Creatinine ratio (ACR) was also categorised representing the clinical categories of risk (no albuminuria, microalbuminuria and macroalbuminuria). Two-way interactions between age with insulin use, as well as between ethnicity with BMI, HbA1c and insulin were tested. Interactions were introduced once the fractional polynomial terms of the main effects were established. The best subset of predictors were selected by performing backward elimination with a stay criterion set to 0.05. To obtain the baseline survival estimate for calculation of the predicted risks, the predicted probability of remaining event-free was evaluated on an individual with zero-valued covariates and a survival time closest to 5 years (this is the mean adjusted baseline risk due to mean centering).

#### Reduced model (model 2)

A reduced model was derived by removing predictors that would require complex tests and expertise for diagnosis and therefore unlikely to be applied in countries where these tests are unaffordable. Therefore, STDR and CVD were removed as predictors. We checked whether the previously insignificant predictors became significant to re-enter the model. This is the first of the simplified models.

#### Minimal-resources model (model 3)

We then derived the minimal-resources model to only include predictors that required simple, and affordable tests so that they can be applied in very resource-constrained countries. Laboratory tests eGFR and ACR were kept in all the models due to their importance in the diagnosis and management of CKD and to emphasise the importance of these routine tests that are relatively inexpensive. Other relatively less predictive and costly laboratory tests were removed (HbA1c and HDL). This is the final simplified model.

### Sensitivity analyses

First, in order to assess the impact of missing data, new prediction models were developed to assess change in discrimination and calibration of models from the original complete-case analysis. For this, we reduced the proportion of missing data in ACR by increasing the criteria for measurement of ACR to within 2-years prior to, or up to 6 months after baseline. Second, TRIPOD guidelines^29^ recommend the assessment of model performance in key subgroups, so we assessed discrimination and calibration in different subgroups in the Wales cohort, including non-modifiable risk factors of age at baseline (< 65 vs >  = 65 years), duration of diabetes at baseline (0 vs > 0 years) and gender both in original models and re-calibrated models (re-calibrated baseline survival in the total cohort). Furthermore, predicted and re-calibrated risks were assessed against observed rates by eGFR categories.

### Internal validation

Model discrimination on the study cohort was assessed using Harrell’s C-statistic^30^. In order to minimise the risk of overfitting bias, internal validation was assessed using tenfold cross-validation^31–34^. The model’s calibration slope was assessed by calculating β coefficient for the linear predictor(LP), which averages the calibration slope across all time points. To assess the separation in risk thresholds, 4 risk groups were determined using the 16th, 50th and 84th centiles of the LP as per Cox’s method^35,36^. Visual separation of the risk groups was assessed using the Kaplan–Meier (KM) plot.

### External validation

Model discrimination and calibration was assessed using Harrell’s C-statistic and the calibration slope, respectively. The LP was categorised using cut-points from model development and event rates were compared between cohorts within each respective risk group. The beta coefficient for the calibration slopes gives an impression of whether risks were over or under-predicted across all time-points, however in order to visualise the calibration slope at a single time point, observed and predicted risks were plotted after categorising risks at 5-years into deciles. Sparse (fewer than 5 events) deciles were handled by collapsing the groups. The baseline survival function was then re-calibrated if miscalibration was detected, by assigning an offset term to the LP in the Wales cohort. The re-calibrated baseline survival estimates were provided at 1-year time increments to ensure transparency of reporting and enable further research to utilise these models in predicting across shorter time horizons.

### Utility in clinical decision making

Decision curve analysis was performed in external validation to assess the clinical utility of the models across clinically relevant threshold probabilities^37^. We presented graphical summaries for the net benefit (benefit vs harm), where the model with the greatest net benefit has the most clinical value.

### Presentation of final models for clinical use

The minimal resources risk model was converted into a risk score using Frank Harrell’s regression nomogram command in R, for ease of interpretation. The risk score was presented in both graphical and tabular form. The agreement between the LP estimates based on the multivariable Cox regression model and the LP estimates based on the points system was assessed using weighted (Equal-spacing and Fleiss-Cohen) Kappa, root mean square error (RMSE) and mean prediction error(MPE). Discrimination and calibration applying the points-based model on the external validation cohort was assessed using the c-statistic and calibration slope (supplemented with graphs), to assess the loss of information from simplifying the models into points.

## Results

### Baseline characteristics

Overall, 20,510 participants met our inclusion criteria in the derivation cohort (figure S1) and 13,346 in the Wales cohort (figure S2). Table 1 shows the baseline characteristics of participants at study entry in both London and Wales cohorts. The validation distribution of characteristics in the Wales cohort overlapped that of the London cohort and were on average comparable in many characteristics. They were on average four years older, with higher BMI, higher proportion of cardiovascular morbidity at baseline, with a higher proportion of male participants, higher proportion with recently diagnosed diabetes and lower proportion of Black ethnicity. The proportion of missing data overall and by missing in ACR (constituting the highest proportion missing, 50%) for the London cohort are provided in tables S2-S4. There were no marked differences in baseline demographic and clinical characteristics used in the models between missing and non-missing participants, indicating low likelihood of violation of the missing at random assumption made in modelling.

**Table 1:** Baseline characteristics of the study cohort.

Patient characteristic	London N=20,510 (events=1378) Mean (±SD) or Median(IQR) or n (%)	Wales N=13,346 (events=656) Mean (±SD) or Median(IQR) or n (%)
Age at baseline (Mean- years)	53.1 (12.7)	57.4(12.4)
Age at diagnosis of T2D (Mean-years)	49.7(12.2)	56.1(12.4)
Gender Male	11,388 (55.5%)	8,591(64.0%)
Ethnicity Black Other	3,820(18.6%) 16,690(81.4%)	171 (1.3%) 13,175(98.7%)
Systolic Blood Pressure (Mean SD– mm Hg)	131.5 (16.8)	136.7 (17.2)
Hypertension (antihypertensive use or SBP>130)	14,425 (70.3%)	10,778 (80.8%)
BMI‡ (Mean SD– kg/m^2^)	30.0 (6.1)	33.4 (7.1)
Duration of diabetes Median IQR - years	0.0(0.0–5.6)	0.0 (0.0, 0.0)
Duration of diabetes (categorical), years ≥ 0 and<2.5 ≥ 2.5 and <5 ≥5 and <10 ≥10	13,249 (64.6%) 1,733 (8.5%) 2,943 (14.4%) 2,585 (12.6%)	11,563(87%) 219 (1.6%) 799 (6.0%) 765 (5.7%)
Cardiovascular morbidity ¥ Yes	2,059 (10.0%)	2,281 (17.0%)
STDR	267 (1.3%)	198 (1.5%)
On insulin Yes	1,441 (7.0% overall or 16.5% in prevalent diabetes cases)	242 (1.8% overall or 12.9% in prevalent diabetes cases)
On anti-HT agents ± Yes	10,542 (51.4%)	7,721 (58.0%)
HbA1c (Median IQR- mmol/mol)	58.5 (50.8–76.0)	56.0 (49.0, 76.0)
HDL-cholesterol (Median IQR – mmol/L)	1.1 (1.0–1.3)	1.10 (0.90, 1.30)
ACR Median IQR – mg/mmol	1.0 (0.5–2.8)	0.90 (0.50, 2.10)
eGFR (Median IQR- ml/min/1.73m2)	87.0 (76.0–90.0)	85.8 (76.0, 97.8)

*T2D* type-2 diabetes; *eGFR* estimated glomerular filtration rate; *Anti-HT* anti-hypertensive; *CVD* cardiovascular disease; *HbA1c* haemoglobin A_1c_; *STDR* sight-threatening diabetic retinopathy; *BMI* Body Mass Index; *HDL* High-density Lipoprotein; *ACR* Albumin: Creatinine ratio; *IQR* Interquartile range.

¥CVD includes Myocardial Infarction (MI), stroke, Atrial Fibrillation (AF), Heart failure (HF), Coronary Heart Disease (CHD) and Peripheral Vascular Disease (PVD).

± Anti-hypertensive agents include Angiotensin receptor blocker/Angiotensin-converting enzyme (ARB /ACE) drugs.

^‡^74 missing BMI in SAIL cohort.

### Incidence of Stage 3 CKD

Incidence of Stage 3 CKD and mean follow-up times of participants are shown in Table S5. Over 5 years of follow-up, 1,378 individuals developed the outcome, corresponding to an incidence of 21.1 per 1000 person-years and stage 3 CKD probability of 0.094 (0.089–0.099) in the London cohort. In Wales, by 5-years 656 events were identified, corresponding to an incidence of 13.2(95% CI; 12.3–14.2) per 1000 person-years and stage 3 CKD probability of 0.068 (0.062–0.073 ). Across all age groups, incidence rates in Wales were systematically lower than that of London, owing to the large proportion (11,474(86%)) of newly diagnosed diabetes cases at baseline in Wales.

### Identified predictors

**Figure 1:**
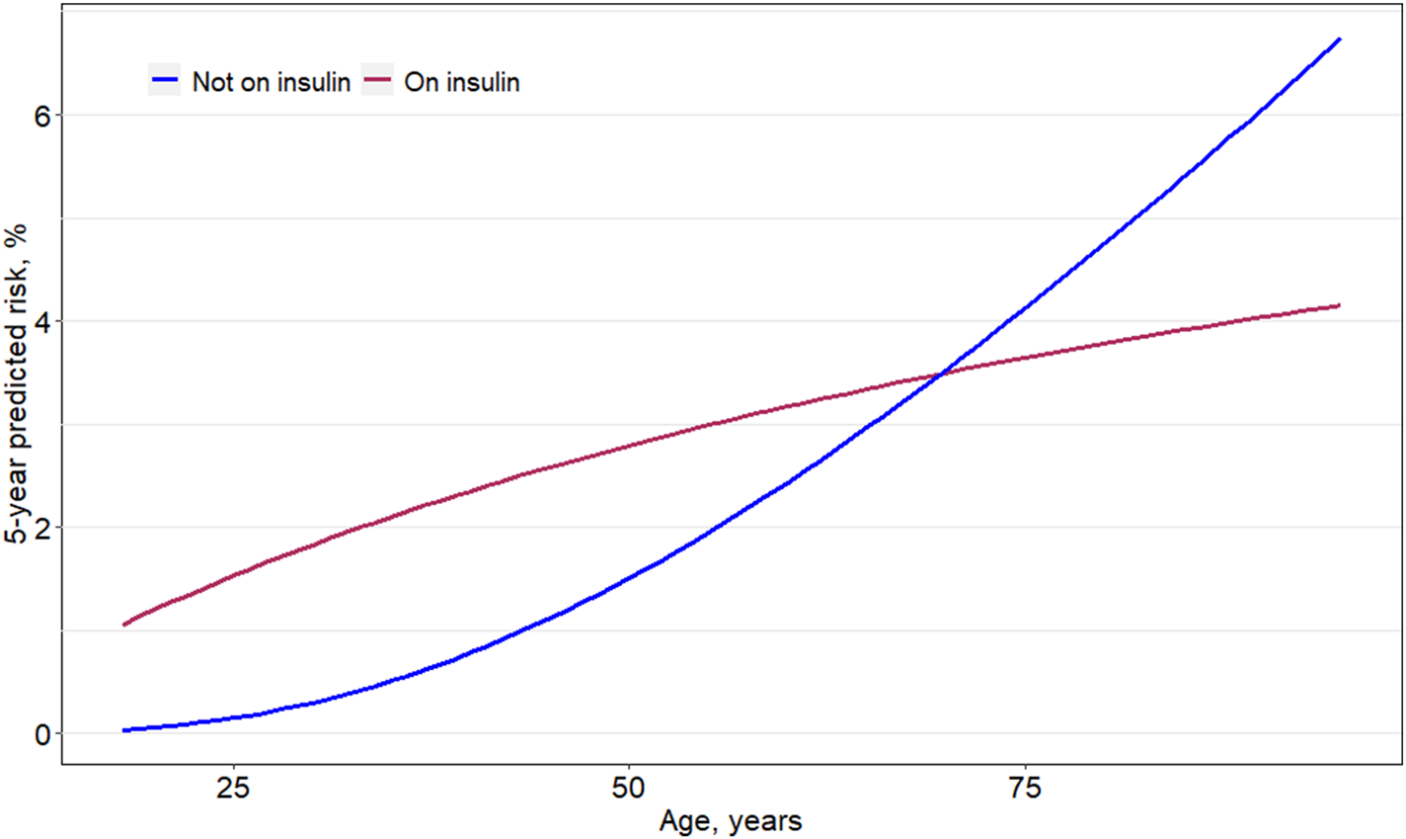
Fractional polynomial terms for age by use of insulin in derived minimal-resources risk model. Interaction effect between age and insulin use in the minimal-resources model, with age modelled as a fractional polynomial term. The y-axis shows the predicted 5-year risk holding all other covariates at the reference value.

Table 2 shows the hazard ratios estimated using the three multivariable Cox regression models from the London cohort. Older age, Sex (male), longer duration of diabetes, higher HbA1c, lower HDL, hypertension, taking anti-hypertensive medication, taking insulin, CVD history, presence of STDR, albuminuria and lower eGFR were significantly associated with incident stage 3 CKD. Age was best modelled using a fractional polynomial term, as shown in Fig. 1 and therefore omitted from Table 2. There was a significant interaction found between age and insulin-use (Fig. 1; *p* < 0.001). The model coefficients remained largely unchanged despite removing several variables from each of the simplified models. Sex was retained in all models as a conceptual confounder for eGFR, despite *p* > 0.05.

**Table 2:** Hazard ratios of selected risk factors for predicting 5-year risk of stage 3 CKD in all three resource-driven models.

Confirmed eGFR < 60 mL/min per 1.73m^2^ (at two time points), stage 3 + code or dialysis (N = 20,510; Events = 1378, total time at risk = 75,420.8 years)									
Patient characteristic	Full model			Reduced model			Minimal-resources model		
	HR	*p*	95% CI	HR	*p*	95% CI	HR	*p*	95% CI
Gender Female Male	– 0.88	– 0.025	– 0.79–0.98	Ref 0.89	– 0.043	– 0.80–1.00	Ref 0.94	– 0.274	– 0.85–1.05
Ethnicity Black†	1.23	0.001	1.09–1.39	1.22	0.002	1.08–1.38	1.18	0.008	1.04–1.33
Duration of diabetes, years	1.05	< 0.001	1.04–1.07	1.05	< 0.001	1.04–1.07	1.05	< 0.001	1.04–1.07
eGFR , mL/min/1.73m2 ≥ 60 and ≤ 70 > 70 and ≤ 80 > 80 and < 90 ≥ 90	13.30 4.48 2.14 Ref	< 0.001 < 0.001 < 0.001 –	10.85–16.31 3.63–5.54 1.69–2.71 –	13.41 4.51 2.15 –	< 0.001 < 0.001 < 0.001 –	10.94–16.44 3.65–5.57 1.70–2.73 –	13.53 4.53 2.16 –	< 0.001 < 0.001 < 0.001 –	11.03–16.58 3.66–5.59 1.71–2.74 –
ACR, mg/mmol No (< 3) Micro (3–30) Macro (> 30)	Ref 1.79 3.36	– < 0.001 < 0.001	– 1.59–2.01 2.72–4.15	Ref 1.80 3.38	– < 0.001 < 0.001	– 1.60–2.03 2.74–4.17	Ref 1.86 3.53	– < 0.001 < 0.001	– 1.65–2.09 2.87–4.35
Systolic blood pressure, per 10 unit increase in mmHg	1.05	0.003	1.02–1.08	1.05	0.004	1.01–1.09	1.05	0.003	1.02–1.08
On Insulin (for age 54 years) No Yes	Ref 1.53	– < 0.001	– 1.24–1.88	Ref 1.59	– < 0.001	– 1.29–1.95	Ref 1.66	– < 0.001	– 1.35–2.03
On AntiHT Agents ±	1.23	0.002	1.09–1.39	1.26	< 0.001	1.11–1.43	1.23	0.001	1.09–1.40
HbA1c, per 10 unit increase in mmol/mol	1.03	0.009	1.01–1.06	1.04	0.008	1.01–1.06	–	–	–
HDL, per 1 unit increase in mmol/L	0.76	0.002	0.63–0.91	0.76	0.003	0.64–0.91	–	–	–
Presence of STDR No Yes	Ref 1.47	– 0.011	– 1.09–1.98	–	–	–	–	–	–
CVD history ¥ No Yes	Ref 1.17	– 0.026	– 1.02–1.34	–	–	–	–	–	–

*T2D* type-2 diabetes; *eGFR* estimated glomerular filtration rate; *Anti-HT* anti-hypertensive; *CVD* cardiovascular disease; *HbA1c* haemoglobin A_1c_; *STDR* sight-threatening diabetic retinopathy; *BMI* Body Mass Index; *HDL* High-density Lipoprotein; *ACR* Albumin: Creatinine ratio.

Models included fractional polynomial terms for age and duration as follows:

Age term was (age/10)^-0.5–0.43 (centered at age 54 years).

Duration term was ln(duration + 0.003)/10 + 1.08.

The models also included interaction terms between age and insulin-use for all three models, omitted from the table.

The effect of the continuous predictor age by insulin-use is shown in Fig. 1 and the model equations presented in further detail in table S6.

¥CVD includes Myocardial Infarction (MI), stroke, Atrial Fibrillation (AF), Heart failure (HF) ,Coronary Heart Disease (CHD) and Peripheral Vascular Disease (PVD).

± Anti-hypertensive agents include Angiotensin receptor blocker/Angiotensin-converting enzyme (ARB /ACE) drugs.

^†^Compared to patients in the White, South Asian, Mixed or Other groups.

### Discrimination

Measures of discrimination and calibration were provided in Table 3. tenfold cross-validated Harrell’s C-statistic remained high with values ranging 0.852–0.853 in all models (Table 3). C-statistics remained high in external validation ranging 0.823–0.826 across models (Table 3).

**Table 3:** Validation statistics for 5-year predicted risk of incident stage 3 CKD.

			Full model	Reduced model	Minimal-resources model	Points-based model ±
Internal validation	London cohort (N = 20,510; events = 1378; total time at risk = 75,420.8 years)	Unadjusted (C-statistic)	0.855(0.846–0.864)	0.855(0.846–0.863)	0.853(0.845–0.862)	0.853(0.844–0.862)
		tenfold cross-validated C-statistic (SD)	0.853 (SD 0.010)	0.853 (SD 0.010)	0.852 (SD 0.010)	0.852 (SD 0.016)
		tenfold cross-validated calibration slope (SD)	0.988 (SD 0.043)	0.989 (SD 0.041)	0.990 (SD 0.044)	1.002 (SD 0.070)
		LR	2568	2556	2539	2536
		HR ¥: Group 2 vs 1 Group 3 vs 1	8.38 (6.58,10.67) 41.47 (32.84,52.36)	8.10(6.38,10.27) 39.57(31.46,49.76)	7.53 (5.97,9.50) 37.60 (30.09,46.99)	7.61(6.02–9.61) 37.81(30.22–47.31)
		LP (mean SD)	0.774 (SD 1.499)	0.771 (1.502)	0.808 (SD 1.487)	0.733 (SD 1.465)
External validation	Wales (N = 13,346; events = 656; total time at risk = 49,352.5 years)	C-statistic	0.827(SE 0.007)	0.826(SE 0.007)	0.824(SE 0.008)	0.823(SE 0.008)
		Calibration slope	1.024(SE 0.035)	1.031(SE 0.035)	1.021(SE 0.035)	1.032(SE 0.036)
		O/E	0.799	0.811	0.810	0.856
		Re-calibrated O/E	0.994	0.992	0.995	1.056
		LR	1000	996.7	979.9	976.6
		HR ¥: Group 2 vs 1 Group 3 vs 1	5.53(4.08,7.5) 25.87(19.24,34.78)	5.47(4.05,7.4) 25.83(19.27,34.64)	5.50(4.08,7.41) 25.25(18.87,33.77)	4.96(3.76–6.54) 22.51(17.21–29.46)
		LP (mean SD)	0.875(SD 1.338)	0.862(SD 1.336)	0.889(SD 1.332)	0.818(SD 1.31)

*LR* likelihood-ratio test statistic; *HR* Hazard ratio; *LP* linear-predictor; *O*/*E* observed risk/estimated risk.

Models were internally validated using tenfold cross validation. ¥ Group 1, 2 and 3 indicate participants grouped into the low-moderate, high and very high-risk groups, respectively. ± For the points-based model categories were determined based on cox’s cut points from the minimal resources model. Moreover, the total score (adapted from coefficients of model 3) was fitted as a predictor on its own in a cox regression model for the validation, the reported statistics can be interpreted as measurements of performance.

### Calibration slope

**Figure 2:**
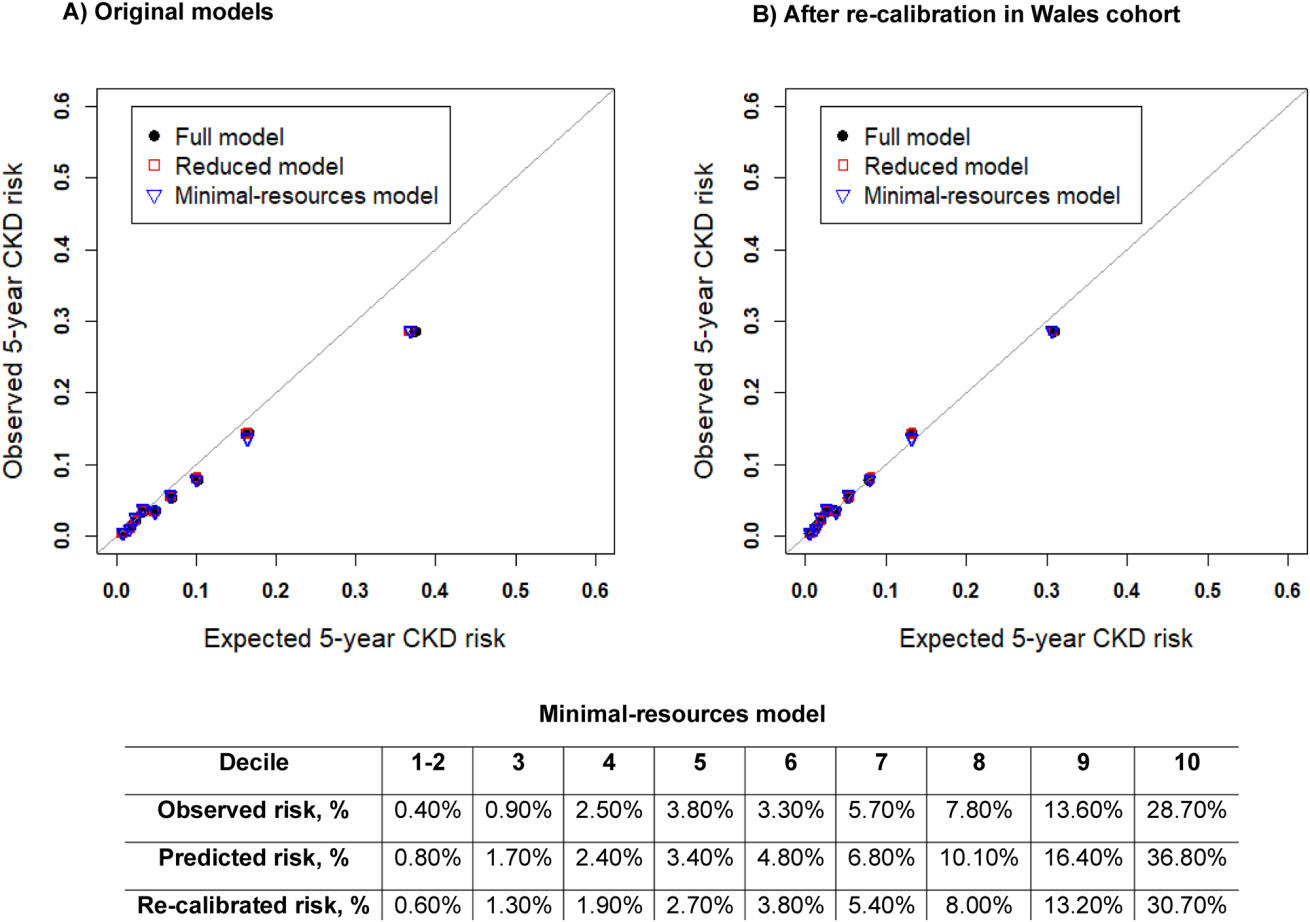
Calibration plots for derived models. Model predictions against observed event rates at 5-years given for 10 risk groups (collapsing first two groups due to inadequate event numbers in first two groups). Predictions from each decile were averaged, so these plots represent mean predicted risk against observed risks (KM-rates). (**A**) Represents 5-year calibration, baseline survival estimates from model development given as; 0.9824, 0.9824, 0.9828 at 5-years for the three models respectively, were used to compute risks in this group. (**B**) Represents 5-year calibration plot- after recalibration, re-calibrated baseline survival estimates given as; 0.9863,0.9860,0.9864 for full, reduced and minimal resources model respectively. Raw risk percentages provided below the plots for the minimal resources model.

tenfold cross-validated calibration slopes measured by the beta coefficient of the LP were all near to 1 in internal validation. The calibration slope in external validation ranged 1.02–1.03 across the three models, showing on average an under-estimation of risk across all time points. The calibration plot (Fig. 2A) was generated after categorising the risks into 10 groups (with 2 groups collapsed due to inadequate number of events) which shows the models are over-predicting risks (O/E ratio ranged 0.799–0.810) at 5-years, particularly in the upper deciles of risk. Following model re-calibration, predicted risks were re-estimated using the recalibrated survival estimate at 5-years in the Wales cohort (given in table S6). In Fig. 2B, the re-calibrated model suggests the predicted risks appear to be better aligned to the observed risks at 5-years, where the upper decile is on average over-predicting by 2% than what is observed. In comparison, the original minimal resources model over-estimates risk by more than 8%. Moreover, O/E ratios in the re-calibrated models are all near to 1 (Table 3).

### Risk groups

The mean(SD) LP shows the relatedness of the samples, these were presented in Table 3. For the validation cohort, we found an increased average LP and a decreased spread of the LP, consistent with deterioration of discriminative ability in the validation cohort.

**Figure 3:**
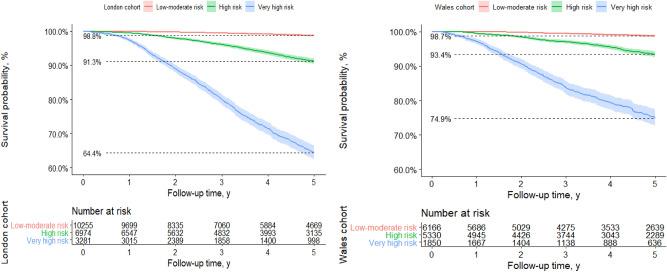
Kaplan–Meier curves stratified by risk groups according to model predictions. This figure shows observed probabilities over time in the derivation and validation datasets by risk group according to predictions from the minimal resources model. The prognostic groups were defined by categorising the linear predictor according to the cut-points representing the 50th and 84th centiles, equivalent to LP(linear predictor) values of: ≤ 0.72, > 0.72 and ≤ 2.36, > 2.36 or predicted risks of ≤ 3.6%, > 3.6% and ≤ 17.2%, > 17.2%.

The adjusted baseline survival estimates were computed from the derivation cohort and were combined with the LP to formulate risk equations (table S6). The survival rates in the three risk groups generated from 50 and 84th centiles of prognostic index applying the minimal resources model are shown in Fig. 3 and labelled as low-moderate risk, high-risk and very high-risk. Incidence rates in these groups were 1.2%, 8.7% and 35.6% respectively (*p* < 0.001; log rank test) compared to an overall KM-rate of 9.4%. This shows how the models through screening will enrich the proportion of positive predictive cases relative to not using this model. The hazard ratios between risk groups (Table 3) were reasonably well-maintained but larger in London for the high-risk group (London; 7.53(95% CI 5.97,9.50) vs Wales 5.50(95% CI 4.08,7.41)), meaning participants in the high-risk group in London develop stage 3 CKD at more than 7 times the rate per unit time compared to the low-risk group. Similarly, the hazard ratios appear larger in the London cohort for the very-high risk group (London; 37.60 (95% CI 30.09, 46.99) vs Wales 25.25(95% CI 18.87,33.77), consistent with over-estimation seen in the calibration of the original models (Fig. 2A).

### Sensitivity analyses

During the model development process, when taking ACR measurements within 2 years before, or within 6 months after baseline, we increased our cohort by an additional 5,729 patients, who had missing ACR when the original 6-month criterion was applied. Previously non-significant covariates (from complete-case analysis of 20,510 samples) did not enter the models. There were no marked differences in multivariable hazard ratios or C-statistics for the models derived from sensitivity analysis to original models (table S7). Model discrimination and calibration of our final three models were assessed in key subgroups in the Wales cohort (table S8). C-statistics and calibration slopes appeared similar and did not differ greatly based on gender, duration of diabetes or age. The calibration slopes in sub-group analysis in all but females and participants above the age of 60 years, suggested an under-estimation of risks across all time-points. In participants 60 years and over, model discrimination averaged 0.77(SE 0.01) across models and predictions were on average over-estimated (calibration slope < 1) across all time points. The calibration plot also indicated poorer calibration for predicting risk at 5-years, and over-estimation of risk was more apparent in the upper risk groups (figure S3), like the analysis on the total cohort. The models were better calibrated to females, across all time points (beta coefficient of calibration slope ranging 0.992–1.00) and at 5-years (figure S3). Following re-calibration of the baseline survival function, calibration plots in various subgroups show an improvement in the calibration slope (figure S4). Moreover, following re-calibration observed rates of incident stage 3 CKD in the baseline eGFR categories were better aligned to the predicted risks (figure S5).

### Clinical utility of models

Figures S6 presents the decision curves of our final models in terms of net benefit in the external validation cohort. Model 3 that requires the least resources can stratify patients with an eGFR at baseline of at least 90 mL/min/1.73m2 to a range of predictions (0%-46%) at 5-years. There was negligible difference in the net benefit of our final models, suggesting that they have equal clinical value. At a threshold probability of 10% the minimal resources model in the Wales cohort identified 6.0 more cases per 100 patients without increasing the number treated unnecessarily and is equivalent to 54 fewer false positives per 100 patients compared to treating everyone.

### Model presentation and recommended model

Predicted risks for the minimal-resources model were converted into “points” assigned to each predictor (table S9) and 5-year predicted risks (and re-calibrated risks) can be ascertained from table S10 after calculating the “total points”. The risk-score was supplemented with graphs presenting total points against predicted probabilities for both the original model and after recalibration (figure S7). Alternatively, the total points can be mapped onto the LP using the following linear transformation: LP =  − 4.34 + 0.05*Total points, which can be used to compute the stage 3 CKD probability. Due to the loss of information from using a points-based system with values of the continuous predictors and points presented to the nearest integer, we assessed the degree of agreement between these two methods. Table S11 illustrates the agreement between 5-year risk estimates produced by the nomogram (points) and those based on evaluating the cox model directly, yielding excellent agreement with minimal loss of information from simplifying models into a risk score (Equal-spacing kappa (κ) = 0.958 (ASE 0.001), Fleiss-Cohen kappa (κ) = 0.976 (ASE 0.001), RMSE = 0.039, MPE = -0.953). The points-based system was evaluated in the Wales cohort and had similar discrimination and calibration compared to the original models (C-statistic; 0.823,SE 0.008 and calibration slope; 1.032,SE 0.036). The calibration slope at 5-years show a similar calibration and re-calibration profile to that of the estimates based on evaluating cox model directly (figure S8). We would therefore recommend the points-based minimal resources model for global use.

## Discussion

In this study we developed 3 (resource-driven) risk models for predicting the onset of stage 3 CKD in multi-ethnic persons with T2D within an economically and socially deprived region of inner London. All models were rigorously internally validated using tenfold cross-validation re-sampling^38,39^ achieving excellent discrimination and when externally validated to a cohort of participants in Wales of predominantly white ethnicity. The models were successfully updated to generalise over datasets covering Wales as a population with a lower incidence of stage 3 CKD. Eliminating variables such as HbA1c, HDL, history of CVD and presence of STDR, led to negligible differences in model performance of the three models. These new prediction equations therefore could be applied to screen diabetes-related CKD to help target prevention strategies accurately in resource-constrained environments.

Table S1 shows the currently available risk models for identifying Stage 3 CKD, defined by reduced eGFR to 60 ml/min/1.73m^2^. Our models differ from these recent prognostic modelling equations. The Risk Equations for Complications Of type 2 Diabetes(RECODe) study equations for CKD, developed using 9,635 Action to Control Cardiovascular Risk in Diabetes(ACCORD) study participants, achieved good discrimination and variable calibration^40^. A variety of equations were developed to assess different endpoints for CKD in this study, all of which included 5–6 (costly) laboratory tests or examinations that require the presence of a trained professional. Therefore, implementing these in regions with basic laboratory facilities is not practical. Our models have reduced the number of laboratory tests to a maximum of 2 tests that are widely available and eliminated the need for determining the history of CVD or screening for STDR, which are usually not obtainable in LMICs. The risk model by O’Seaghdha^41^ consisted of the least number of resources (eGFR, ACR, diabetes, hypertension and age), developed using data from the Framingham heart study (FHS) and previously externally validated in 1777 participants from the Atherosclerosis Risk in communities(ARIC) Study (C-statistic; 0.79 in Framingham, 0.74 in ARIC). O’Seaghdha et al. presented an even simpler model, however it did not have eGFR or urine ACR which are known clinically important variables used to guide treatment decisions for CKD^4,42,43^. Furthermore, participants from FHS were 100% white and 76% white in the ARIC study. Such equations would have poorer transportability to LMICs or ethnically diverse populations, whereas ours has been shown to validate well in ethnically homogenous (Wales) and heterogenous populations (inner London).

The risk equations by Nelson et al.^44^ developed on 34 multinational cohorts from the CKD Prognosis Consortium, predicts incident eGFR < 60 mL/min/1.73m^2^ with excellent discrimination but again the final study equation required 5 laboratory/examination variables.

A study by Jardine et al.^45^, conducted on 11,140 T2D participants from the Action in Diabetes and Vascular Disease(ADVANCE) trial shows eGFR and urine ACR to be the leading predictors for predicting stage 3 CKD and when taken together yield a similar c-statistic(95% CI) to ours (0.818(0.781–0.855)). Their final risk prediction model which incorporated 7 risk factors produced a C-statistic(95% CI) of 0.847 (0.815–0.880). While model discriminatory power was high and model variables kept to a minimum, the study participants recruited were a select group with high vascular risk and therefore may not generalise to an unbiased population of people with T2D.

The strengths of our study are that we ensured applicability of these risk tools to routine clinical care in LMIC by developing resource-based models. Derivation of our models was based on a large sample, achieving adequate statistical power. Furthermore, the average differences between our cohorts reflect that this is a more challenging external validation of a model. This is an advantage in validation research as these models with good external performance tend to substantiate the transportability of the models over reproducibility^46^. Decision curve analysis revealed negligible differences in terms of net benefit for our final three models. These economically viable risk models have near equal clinical utility, warranting the removal of several commonly used tests in CKD risk models and allowing for more accessible risk equations for use in poorly resourced communities. Furthermore, we have converted a complex statistical model derived from FP functions and interactions into a simple risk score, showing excellent agreement with predictions from direct evaluation of the cox model. Therefore, our most resource-minimal model can be easily applied in routine clinical care for decision support.

However, there were several limitations. Firstly, due to the study design and gradual decline in renal function in mostly asymptomatic persons with diabetes, the exact date of onset of Stage 3 CKD is difficult to pinpoint and so underestimation of the incidence of Stage 3 CKD cannot be ruled out^47^. However, people with T2D undergo regular blood tests unlike people in the general population, and so likelihood of miss-classification bias would be low. Secondly, although discrimination was maintained throughout the models, initial attempts to validate in Wales prior to recalibration show the calibration at 5-years were suboptimal. O/E ratios were below 1, suggesting that the models were over-fitted to the developmental dataset. The predictive performance was improved following recalibration of the baseline survival function, as incidence rates were generally lower in the Wales cohort. Thirdly, risk equations may be redundant if primary care practices do not encourage regular testing for albuminuria, where missing data in ACR was particularly high in both London (50%) and Wales (49%) cohorts. We stress that for identifying incident stage 3 CKD, it would be improper to advocate diabetes risk models without albuminuria or eGFR^4,42,43^. Finally, external validation in LMIC’s and in other clinically relevant sub populations is encouraged to ensure transportability and generalisability of the risk models. This is currently challenging due to the lack of quality primary care data in LMICs.

In conclusion, we have developed and validated three resource driven models in T2D that may be applied globally to predict incident Stage 3 CKD. Our models can be applied for population screening using the least number of costly variables, enabling more efficient detection of people who require urgent prevention strategies. In resource constrained environments we would favour use of the minimal-resources model presented as a risk score, conserving most of the predictive information and consisting of the least number of variables. However, further external validation is recommended especially for utilisation in pragmatic prevention trials on CKD.

## Supplementary Information

Supplementary Information.

## Data Availability

The primary output of this study are the risk models and the equations are in the manuscript. The data used for the study is third-party data: it is held by Queen Mary’s University London (QUML) and the SAIL Databank at Swansea University on behalf of health care providers in Inner London and Wales who are the original data owners respectively. The permission to access fully anonymised previously curated data from QUML was obtained from the Caldicott guardian and data from SAIL is available to anyone via an application to SAIL.

## Abbreviations

FP: Fractional polynomial
LP: Linear predictor
KM: Kaplan–Meier
RMSE: Root mean square error
MPE: Mean prediction error
FHS: Framingham heart study
ARIC study: Atherosclerosis in communities study
ASE: Average standard error
