# Supplementary material for: Development and validation of resource-driven risk prediction models for incident chronic kidney disease in type 2 diabetes
Source: Sci Rep. 2021 Jul 1;11:13654. doi: 10.1038/s41598-021-93096-w (PMC8249456; doi:10.1038/s41598-021-93096-w)
Supplement: Supplementary file 1 — Supplementary Information. [file 41598_2021_93096_MOESM1_ESM.docx]

## **Supplementary file**

# **Development and validation of resource-driven risk prediction models for incident chronic kidney disease in type 2 diabetes.**

*Sarega Gurudas, Institute of Ophthalmology, University College London, London, UK.

*Dr. Manjula Nugawela, Institute of Ophthalmology, University College London, London, UK.

Professor A Toby Prevost PHD, Nightingale-Saunders Clinical Trials and Epidemiology Unit, King’s College London, London, UK.

Dr Thirunavukkarasu Sathish PHD, Department of Primary Care and Public Health, Imperial College London, London, UK

Dr. Rohini Mathur, Institute of Applied Data Science, Queen Mary’s University London, London, UK

Dr. J M Rafferty, Swansea University, Wales, UK

Dr. Kevin Blighe, Institute of Ophthalmology, University College London, London, UK

Dr. Ramachandran Rajalakshmi, Madras Diabetes Research Foundation, Dr Mohan's Diabetes Specialities Centre, Chennai, India

Dr. Anjana R Mohan, Madras Diabetes Research Foundation, Dr Mohan's Diabetes Specialities Centre, Chennai, India

Ms Jebarani Saravanan. Madras Diabetes Research Foundation, Dr Mohan's Diabetes Specialities Centre, Chennai, India

Professor Azeem Majeed, School of Public Health Imperial College London, London, UK

Professor Viswanthan Mohan, Madras Diabetes Research Foundation, Dr Mohan's Diabetes Specialities Centre, Chennai, India

Professor David R Owens, Clinical Professor of Diabetes, Swansea University, Wales, UK

Dr. John Robson, Institute of Applied Data Science, Queen Mary’s University London, London, UK

Professor Sobha Sivaprasad, Professor of Clinical Retinal Research, NIHR Moorfields Biomedical Research Centre and University College London, London, UK

For the ORNATE India Study Group.

### **Figure S1. Derivation of Development Cohort (London Cohort)**

Patients identified with any clinical code for T2DM in the period 2007-2017 (n=105,533)

Records excluded (n=37,808)

- Those that did not have T2DM date (n=472)
- Those who left, died or turned 18 before study start or registered after study end (n=12,297)
- Those without demographic data (age, sex and ethnicity)
  (n = 2,519, only 5 were missing in age, sex )
- Those that did not have one years follow up data, where cohort exit includes date of death, study end and de-registration (does not yet include date of CKD diagnosis) (n=9,612)
- Those without a code for T2DM between cohort entry and exit (n=10,307)
- Recording errors: T2DM onset before d.o.b (n=27)
- Those with T2DM onset before 18^th^ birthday (n=143)
- On insulin prior to T2DM onset (n=1,483)
- DR at T2DM onset (n=387)
- Those with >50 years diabetes duration (n=4)

T2DM patients eligible for the study, with complete demographic data (n=67,725)

Record excluded (n=47,215)

- Missing eGFR or less than 2 records (n=6,098) or Prior history of eGFR < 60 or within 6 months of baseline (n=11,588)
- Those without covariate data within 6 months of baseline date (n=29,529)

T2DM patients free from CKD at baseline, with complete covariate data (n=20,510)

Abbreviations: T2DM- type 2diabetes; CKD -chronic kidney disease; eGFR-estimated glomerular filtration rate; d.o.b, date of birth

### **Figure S2. Derivation of Validation cohort (Wales cohort)**

Patients identified with any clinical code for T2DM in the period 2007-2017 (n=140,157)

Records excluded (n=75,087)

- Those that did not have T2DM date (n=0)
- Those who left, died or turned 18 before study start or registered after study end (n=245)
- Those without demographic data (age, sex and ethnicity)
  (n =70,688)
- Those that did not have one years follow up data, where cohort exit includes date of death, study end and de-registration (does not yet include date of CKD diagnosis) (n= 850)
- Those without a code for T2DM between cohort entry and exit (n=1,336)
- Recording errors: T2DM onset before d.o.b (n=0)
- Those with T2DM onset before 18^th^ birthday (n= 37)
- On insulin prior to T2DM onset (n=1385)
- DR at T2DM onset (n= 389)
- Those with >50 years diabetes duration (n=0)
- Those age <19 & >95 (n= 157)

T2DM patients eligible for the study, with complete demographic data (n=65,070)

Record excluded (n=51,724)

- Missing eGFR or less than 2 records (n=769)
- Prior history of eGFR < 60 or within 6 months of baseline (n=21,960)
- Those without covariate data within 6 months of baseline date (n=28,995)(Those included in final risk models)

T2DM patients free from CKD at baseline, with complete covariate data (n=13,346)

### **Figure S3. Calibration plots at 5-years in various subgroups for all three derived risk models**


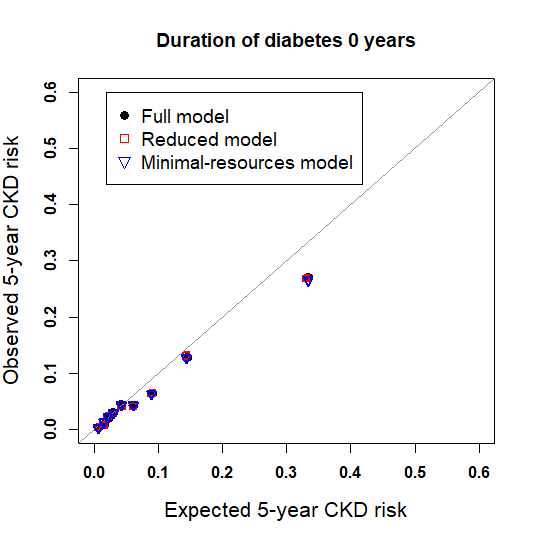

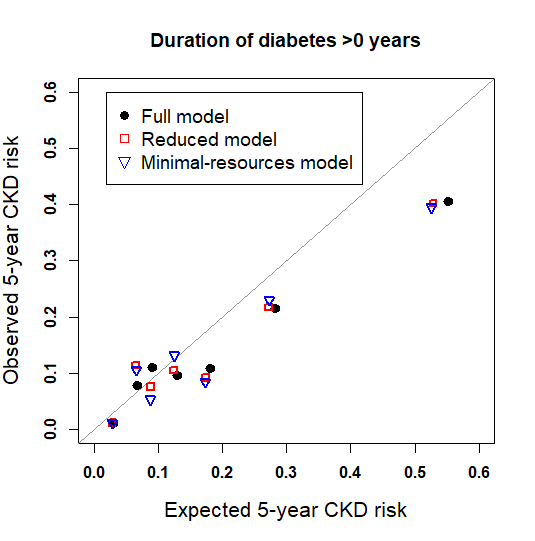

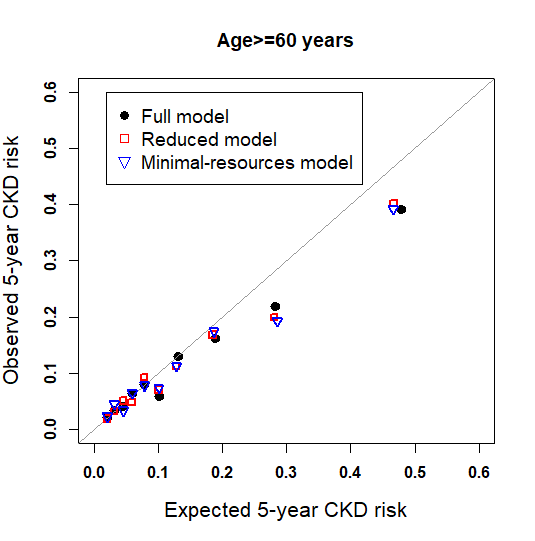

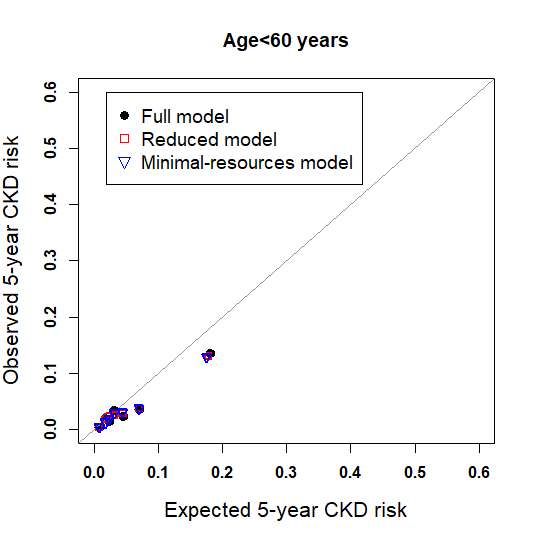

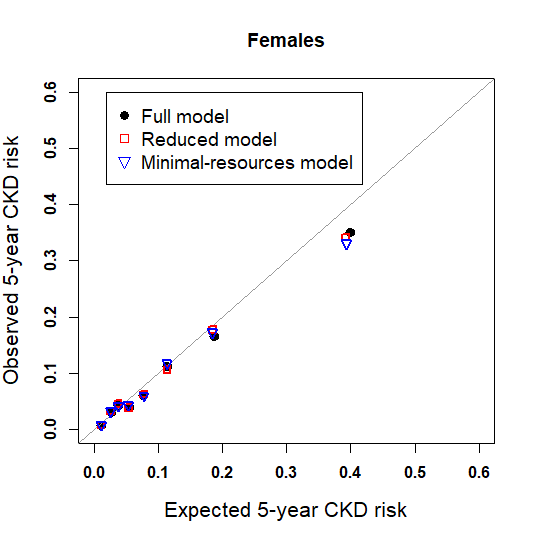

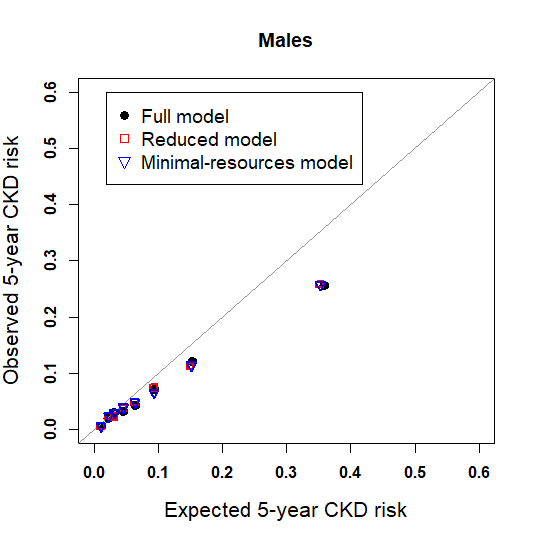


### **Figure S4. Calibration plots at 5-years following re-calibration of baseline survival to whole cohort, in various subgroups**


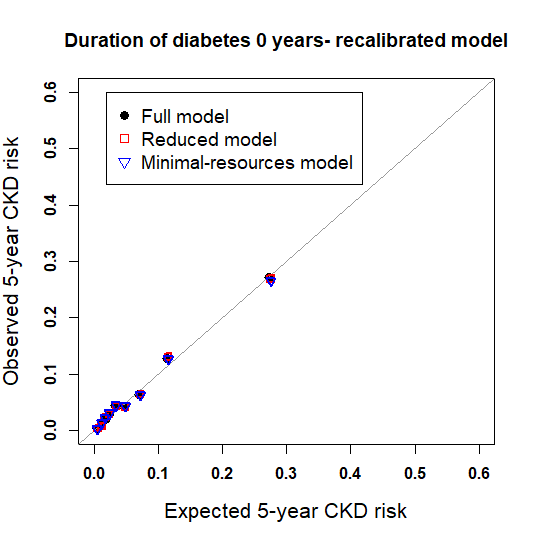

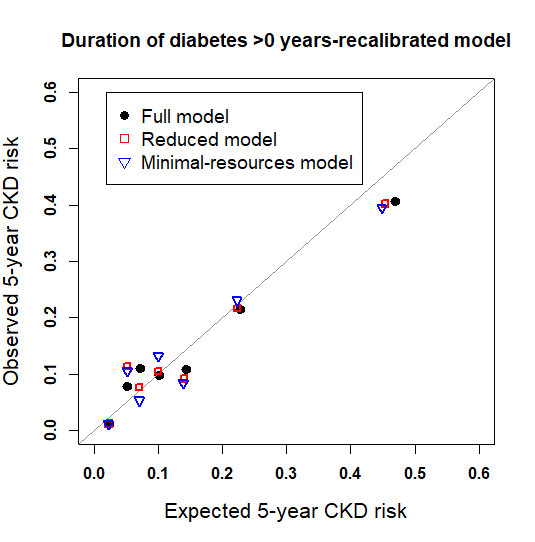

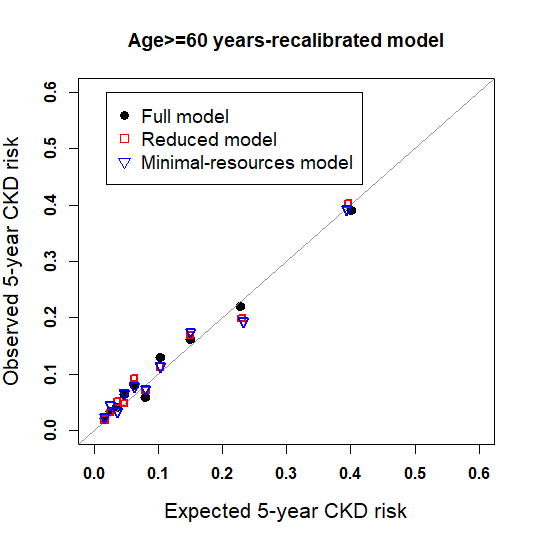

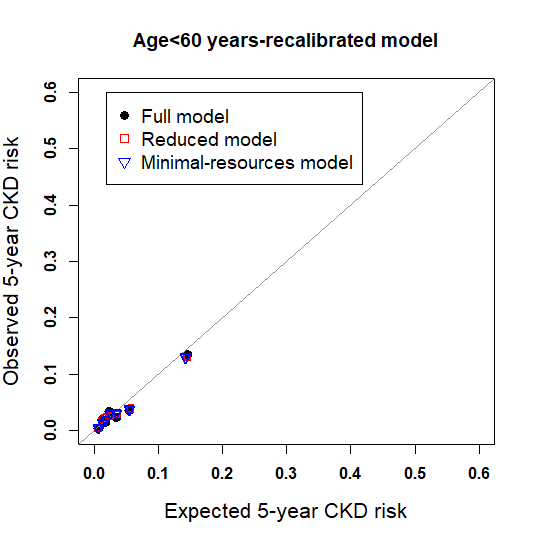

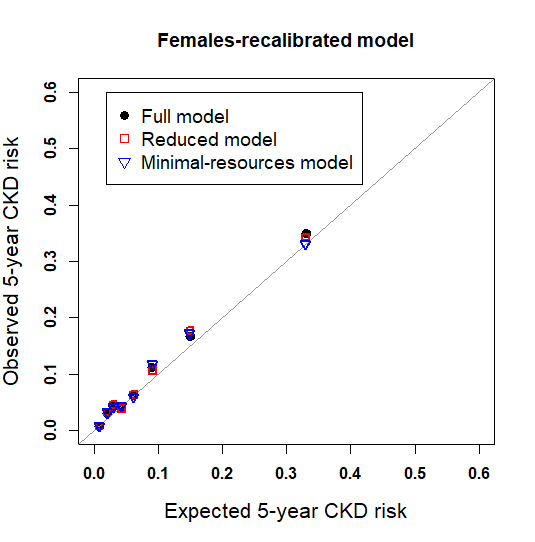

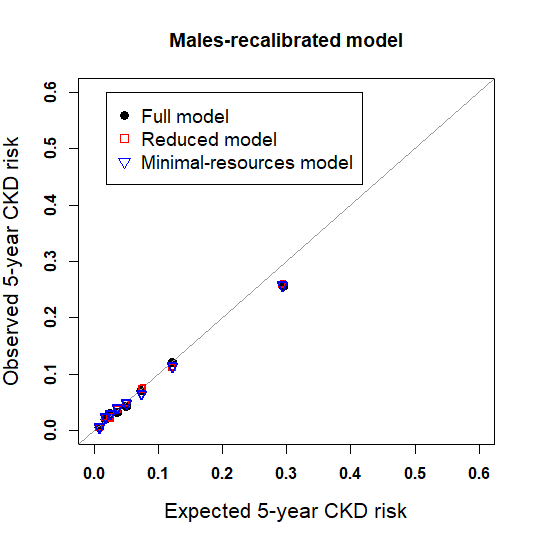


Re-calibrated risks based on re-calibrated baseline survival estimate shown for various subgroups. Re-calibrated baseline survival function evaluated at 5-years, estimates provided in table 6.

### **Figure S5. Calibration and re-calibration by eGFR categories**


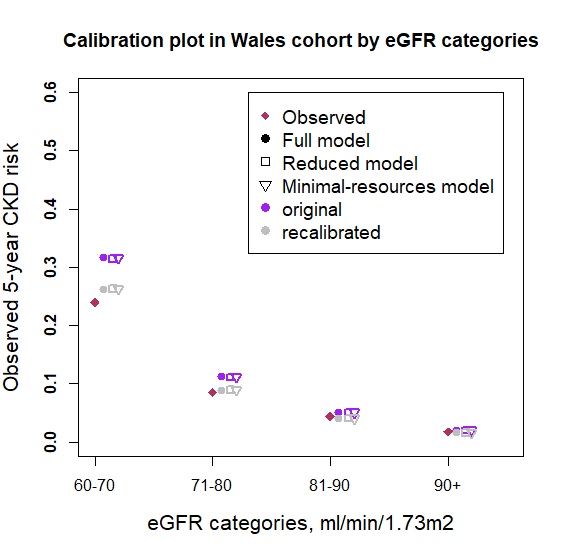


| **Minimal-resources model** | | | |
| --- | --- | --- | --- |
| **Category** | **Observed risk, %** | **Predicted risk, %** | **Recalibrated risk, %** |
| **60-70** | 24.0% | 31.8% | 26.1% |
| **71-80** | 8.5% | 11.3% | 8.9% |
| **81-90** | 4.4% | 5.2% | 4.1% |
| **90+** | 1.8% | 2.0% | 1.6% |

Calibration plot in eGFR categories with observed and predicted and recalibrated risks based on re-calibrated baseline survival estimate presented. Raw risk percentages provided for the minimal resources model

###

### **Figure S6. Decision curves for predicting 5-year risk of incident CKD comparing models in external validation cohort**


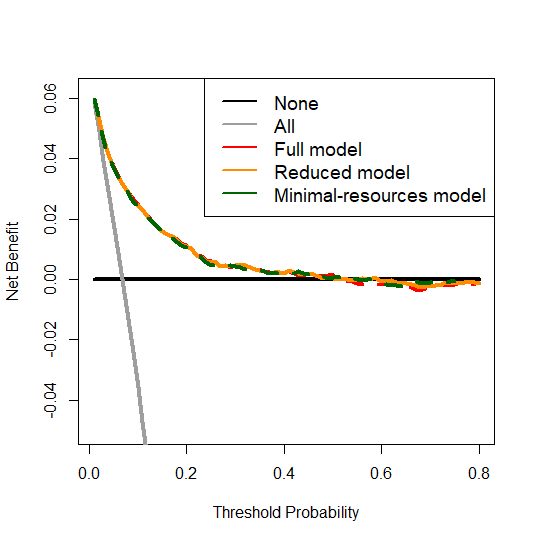


This graph shows the expected net benefit for each threshold probability evaluated from 0 to 80% for the 5-year risk of incident CKD relative to screening no one in the population, i.e. “ None” (black line). Grey line or “All”: Screening everyone in the population. Screening patients by applying threshold probabilities to the model predictions from the: “Full model” (Red line), “Reduced model” (Orange line), “Minimal-resources model” (green line).

Net benefit is defined by $net benefit= ((True positive/N) - (False positives/N) x( pt/(1-pt));$ where p_t_ is the probability threshold ).True positive count and false positive count defined by $\# True positives = \left[ 1-s\left( t \right) | z=1 \right]*P\left( z=1 \right)* n$ and $\# False positives = \left( s\left( t \right) | z=1 \right)* P\left( z=1 \right)x*n$, where s(t) is the survival probability at time t, z is an indicator variable taking value 1 if the predicted probability for the patient >= p_t_ .

The reduction in the number of false positives per 100 individuals under each model is calculated using the following formula:

$reduction in the number of false positives per 100 individuals=(net benefit of the model-net benefit of treat all)/(pt/(1-pt)) x 100.$

**Figure S7. Risk score interpretation (5-year predicted risks)**


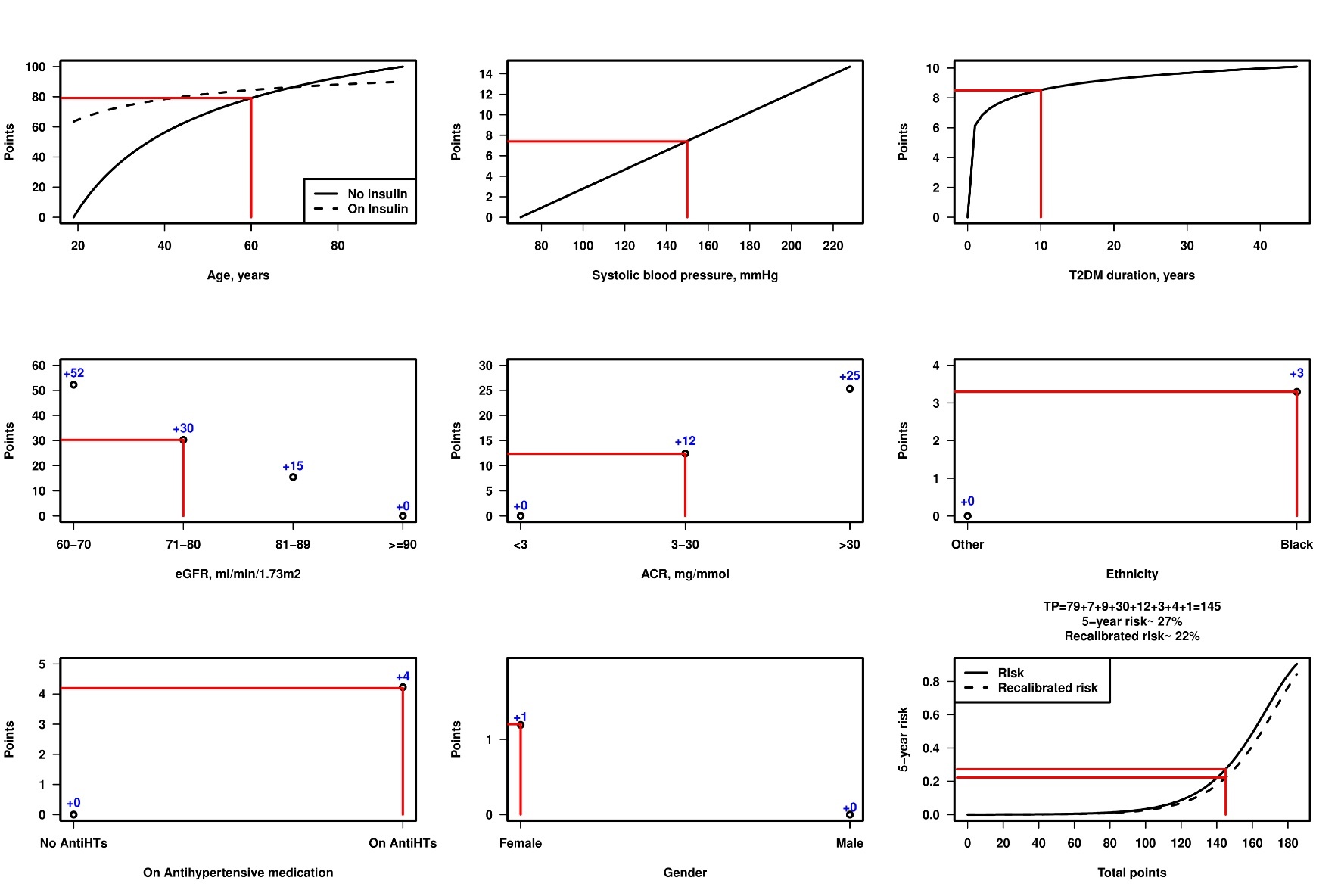


Figure presenting the minimal-resources risk score (points-based model), showing points for each predictor and a translation of total points to predicted probabilities of stage 3 CKD. Example patient marked in red; Age 60 years, SBP 150 mmHg, T2DM duration 10 years, eGFR 71-80 ml/min/1.73m2, ACR 3-30 mg/mmol, Black ethnicity, On AntiHT’s and female gender. Points were rounded to the nearest whole number. In tabular form, the risk score and translation of total points to predicted probabilities given in tables S9 & S10 respectively

### **Figure S8. Calibration plot in Wales for points-based model**


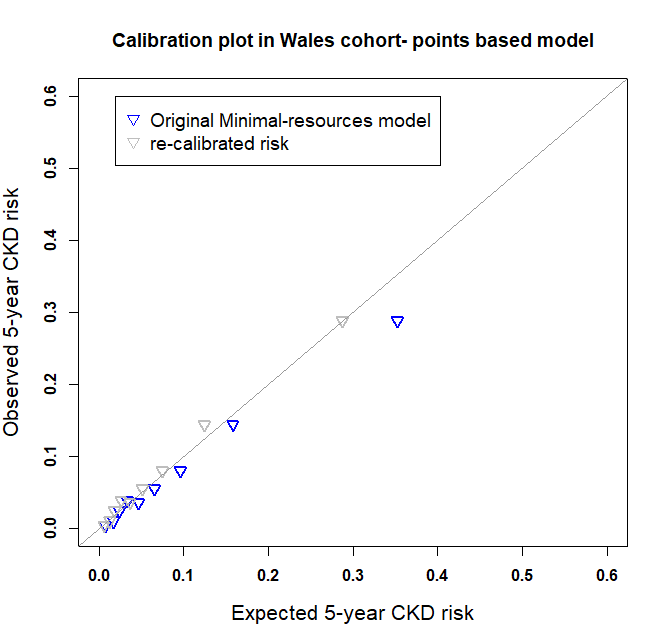


Model calibration and re-calibration for minimal-resources model when coverted into points using a regression nomogram. These plots can be compared to calibration plot in figure 3 to show similar 5-year calibration when evaluating risks using the cox model directly to using the risk-score.

### **Table S1. Recent risk prediction models and variables used for use in low- and middle-income countries**

| **Study – First Author/Year** | **Population** | **Outcome** | **Variables used** | **Number of invasive/difficult to obtain/costly variables in simplest model** | **Ethnic diversity** |
| --- | --- | --- | --- | --- | --- |
| Nelson/2019[1] | 34 multinational general population cohorts (N=4,441,084 participants without diabetes and N=781,627 participants with diabetes) | eGFR< 60 ml/min/1.73 m^2^ at two points and other signs of kidney disease including dialysis or transplant | Age, Gender, Black race, eGFR, CVD, Smoker, Hypertension, BMI, ACR, HbA1c | 4 | 34 multi-national cohorts: White race ranging 0%-100%, black race 0% -100%, Asian 0%-100%, Hispanic race 0%-37% |
| Basu/2017[2] | Action to Control Cardiovascular  Risk in Diabetes study(ACCORD) ( N=9635; 2001–09) | Doubling of  serum  creatinine or  >20 mL/min  per 1・73 m²  decrease in  eGFR | Age, Gender, Ethnicity, smoker, SBP, CVD, Anti-HT, OAD, anti-coagulants, HbA1c, HDL, creatinine, ACR | 5 | 19% black, 7% Latino or Hispanic (derivation cohort, ACCORD); 24% black, 17% Latino (DPPOS); 16% black and 14% Latino (Look AHEAD) |
| Mise/2016[3] | Japanese with T2DM and biopsy proven DKD (N=149 individuals) | Decline in eGFR of ≥ 50% from baseline | urinary N-acetyl-β-d-glucosaminidase, urinary β2-microglobulin (β2-MG), interstitial fibrosis and tubular atrophy (IFTA) score, age, sex, body mass index, diabetic retinopathy, systolic BP , urinary protein excretion, and eGFR at baseline | 7 | Japanese |
| Mocroft/2015[4] | D:A:D study, consisting of individuals from Europe, US and Australia (N=17,954 HIV-positive individuals) | eGFR< 60 ml/min/1.73 m^2^ at two points (at least 3 months apart) | Gender, intravenous drugs, without hepatitis coinfection age, eGFR, CD4 count , hypertension ,prior CVD, diabetes | 4 | 46.6% white, 7.2% black, 2% other, 44.3% unknown |
| O’Seaghdha/2012 [5] | US, Framingham heart study participants (N=2,490, 1995-2008) | Development of eGFR < 60 ml/min/1.73m2 | Model 1: Age, diabetes, hypertension  Model 2: Model 1+eGFR  Model 3: Model 2 +albuminuria | 0 | 100% White |
| Alssema/2012 [6] | 3 population-based cohorts from Netherlands (N=2,840) | Development of eGFR < 60 ml/min/1.73m2 | Age, BMI, waist circumference, antihypertensive treatment, smoking, family history of myocardial infarction or stroke, and family history of diabetes | 2 | 100% White |
| Jardine/2012 [7] | ADVANCE Study participants (N=11,140, 2001-2003) | Doubling of serum creatinine to >=2.26 mg/dl ( >=200 mmol/l), renal replacement therapy, or renal death in diabetes | Gender, Ethnicity, eGFR, ACR, SBP, HbA1c, DR, Age at completion of formal education | 4 | 38% Asian, 59% white, 0.4% black, |
| O’Seaghdha/2011 [8] | US, Framingham heart study participants (N=2,490, 1995-2008) | Development of eGFR < 60 ml/min/1.73m2 | Model 1: Age, sex, genetic risk score  Model 2: Model 1 + baseline eGFR, cohort status, hypertension, diabetes, proteinuria | 1 genetic risk score | 100% White |
| Ando/2011 [9] | HIV infected individuals patients at Tokyo metropolitan Komagome Hospital (N=623, Jan-March 2008) | Development of eGFR < 60 ml/min/1.73m2 | Model 1: HIV exposure, Hepatitis C coinfection, Age, eGFR, Gender, Nadir CD4 count  Model 2:  Model 1+ Hypertension, CVD, Diabetes | 2 | 100% Japanese |
| Fox/ 2010 [10] | participants who attended the sixth Framingham Offspring Study examination (N=2345 ,1995 to 1998) | Development of eGFR < 60 ml/min/1.73m2 | Model 1: Age and gender  Model 2: Model 1+ systolic blood pressure, hypertension treatment, smoking, body mass index, HDL, diabetes, baseline eGFR (for incident CKD)  Model 3:  Model 2+ homocysteine and aldosterone | 0 | 100% White |
| Hippisley-Cox/2010 [11] | QResearch cohort (N= 1,591,884) | Development of eGFR < 60 ml/min/1.73m2 | age, ethnicity, deprivation, smoking, BMI, systolic blood pressure, diabetes, rheumatoid arthritis, cardiovascular disease, treated hypertension, congestive cardiac failure; peripheral vascular disease, NSAID use and family history of kidney disease. | 7 | 95.5% White or not recorded; 1.7% Indian, Pakistani, Bangladeshi or Other Asian; 1.28% Black; 0.18% Chinese; 0.88% Other |
| Chein/2010 [12] | Taiwan/Chinese population (N=5168) | Development of eGFR < 60 ml/min/1.73m2 | Model 1: age, BMI, diastolic blood pressure, history of type 2 diabetes and stroke  Model 2: Model 1+ uric acid ,postprandial glucose, haemoglobin A1c, and proteinuria 100 mg/dL or greater | 2 | 100% Taiwan/Chinese (Asian) |
| [Kshirsagar](https://pubmed.ncbi.nlm.nih.gov/?sort=pubdate&term=Kshirsagar+AV&cauthor_id=19064831)/2008[13] | Combined cohort of the Atherosclerosis Risk in Communities Study and the Cardiovascular Health Study (N = 14 155) | Development of eGFR < 60 ml/min/1.73m2 | Model 1:  age, anaemia, female sex, hypertension, diabetes mellitus, peripheral vascular disease, and history of congestive heart failure or cardiovascular disease  Model 2: Model 1 + ethnicity ,HDL | 3 | 21% non-white |

Recent risk prediction model development studies. Inclusion criteria: prospective/retrospective cohort studies that have incorporated diabetes as a risk factor or models developed exclusively on diabetes participants, models must present a study equation and perform an assessment of discrimination and calibration, outcome must pertain to CKD defined by reduced eGFR. Cross-sectional studies have not been included for comparison as they study classification of prevalent CKD rather than prediction of CKD incidence. ‘Number of invasive/difficult to obtain/costly variables in simplest model’ column include variables that require blood test or diagnostic test such as biomarkers, cardiovascular disease assessment, diabetic retinopathy screening examination, genetic testing etc

### **Table S2. Missing data; Missing data at baseline for London and Wales cohorts**

| **Patient Characteristic** | **N(%) London**  **N=50,039** | **N(%) Wales**  **N=42,341** |
| --- | --- | --- |
| **Systolic Blood Pressure**  mm Hg | 1,218 (2.4%) | 2,966 (7.0%) |
| **BMI**  kg/m^2^ | 3,930 (7.9%) | - |
| **HbA1c**  mmol/mol | 3,255 (6.5%) | 14,929 (35.3%) |
| **Total Cholesterol**  mmol/L | 3,463 (6.9%) | - |
| **HDL cholesterol**  mmol/L | 7,034 (14.1%) | 8,257 (19.5%) |
| **Albuminuria**  mmol/ml | 25,143 (50.3%) | 21,056 (49.7%) |
| **eGFR**  ml/min/1.73m2 | 6,569 (13.1%) | 3,678 (8.7%) |

Abbreviations: BMI- Body Mass Index; eGFR-estimated glomerular filtration rate; HbA1c-glycated haemoglobin; HDL -high density lipoprotein

###

### **Table S3. Missing data; Baseline characteristics of complete vs incomplete cases in London**

| **Patient Characteristic** | **Complete cases (N=20,510)** | **Incomplete cases (N=29,529)** |
| --- | --- | --- |
| **Events** | 1,378 (6.7%) | 1,842 (6.2%) |
| **Age at baseline**  Mean (SD) | 53.1 (12.7) | 53.4 (13.1) |
| **Gender**  Female  Male | 9,122 (44.5%)  11,388 (55.5%) | 13,502 (45.7%)  16,027 (54.3%) |
| **T2DM Duration**  Median (IQR) | 0 (0-5.6) | 0 (0-5.8) |
| **Ethnicity**  White  South Asian  Black  Other/Mixed/Missing | 4,493 (21.9%)  11,116 (54.2%)  3,820 (18.6%)  1,081 (5.3%) | 7,615 (25.8%)  14,790 (50.1%)  5,399 (18.3%)  1,725 (5.8%) |
| **Hba1c (mmol/mol)**  Median (IQR) | 58.5 (50.8-75) | 57.4 (50-73) |
| **HDL**  Median (IQR) | 1.1 (1.0-1.3) | 1.1 (1.0-1.4) |
| **Cholesterol**  Mean (SD) | 4.7 (1.3) | 4.7 (1.3) |
| **SBP**  Mean(SD) | 131.5 (16.8) | 131.3 (16.9) |
| **eGFR**  60-70  70-80  80-90  >90  Median IQR | 2,648 (12.9%)  4,360 (21.3%)  4,572 (22.3%)  8,930 (43.5%)  87 (76-90) | 2,968 (12.9%)  4,901 (21.4%)  4,955 (21.6%)  10,136 (44.2%)  87 (77-90) |
| **BMI**  Mean(SD) | 30.0 (6.1) | 30.1 (6.4) |
| **On Insulin**  No  yes | 19,069 (93%)  1,441 (7.0%) | 26,993 (91.4%)  2,536 (8.6%) |
| **On antiHT agents**  No  yes | 9,968 (48.6%)  10,542 (51.4%) | 15,047 (51.0%)  14,482 (49.0%) |
| **Cardiovascular outcomes (including MI, stroke, AF, HF and CHD)**  No  Yes | 18,451 (90.0%)  2,059 (10.0%) | 26,136 (88.5%)  3,393 (11.5%) |
| **STDR** | 267 (1.3%) | 333 (1.1%) |
| **Albuminuria**  No  Micro  Macro  Median IQR | 15,601 (76.1%)  4,388 (21.4%)  521 (2.5%)  1.0 (0.5-2.8) | 3,133 (71.4%)  1,128 (25.7%)  125 (2.9%)  1.3 (0.6-3.6) |

Abbreviations: T2DM- type 2 diabetes; eGFR-estimated glomerular filtration rate; HbA1c-glycated haemoglobin; HDL- high density lipoprotein; SBP-systolic blood pressure; BMI- Body mass index; antiHT- antihypertensive agents; AF-atrial fibrillation; HF-heart failure; MI-myocardial infarction; CHD-coronary artery disease; STDR-sight threatening diabetic retinopathy; SD-standard deviation; IQR-interquartile range.

### **Table S4. Missing data; Baseline characteristics of missing vs non-missing urine ACR in London**

| **Characteristic** | **Urine ACR non-missing (N=24,896)** | **Urine ACR missing (N=25,143)** |
| --- | --- | --- |
| **Events** | 1,569 (6.2%) | 1,649 (6.6%) |
| **Age at baseline** Mean (SD) | 53.2 (12.8) | 53.3 (13.0) |
| **Gender**  Female  Male | 11,028 (44.3%)  13,868 (55.7%) | 11,596 (46.1%)  13,547 (53.9%) |
| **T2DM Duration** Median 25^th^-75^th^ | 0 (0-5.9) | 0 (0-5.6) |
| **Ethnicity**  White  South Asian  Black  Other/Mixed/Missing | 5,712 (22.9%)  13,105 (52.6%)  4,724 (19.0%)  1,355 (5.4%) | 6,396 (25.4%)  12,801 (50.9%)  4,495 (17.9%)  1,451 (5.8%) |
| **HbA1c (mmol/mol)** Median 25^th^-75^th^ | 58.5 (50.8-97.8) | 57.4 (50-72.7) |
| **HDL** Median 25^th^-75^th^ | 1.1 (1.0-1.3) | 1.1 (1.0-1.4) |
| **Cholesterol** Mean (SD) | 4.70 (1.3) | 4.7 (1.3) |
| **SBP** Mean(SD) | 131.5 (16.8) | 131.2 (16.9) |
| **eGFR**  60-70  70-80  80-90  >90  Median 25^th^-75^th^ | 2,970 (12.9%)  4,826 (21.0%)  5,032 (21.9%)  10,173 (44.2%)  87 (76-90) | 2,646 (12.9%)  4,435 (21.7%)  4,495 (22.0%)  8,893 (43.5%)  87 (76-90) |
| **BMI** Mean(SD) | 30.1 (6.2) | 30.1 (6.4) |
| **On Insulin**  No  yes | 23,014 (92.4%)  1,882 (7.6%) | 23,048 (9.7%)  2,095 (8.3%) |
| **On antiHT agents**  No  yes | 12,162 (48.9%)  12,734 (51.2%) | 12,853 (51.1%)  12,290 (48.9%) |
| **Cardiovascular outcomes (including MI, stroke, AF, HF and CHD)**  No  Yes | 22,314 (89.6%)  2,582 (10.4%) | 22,273 (88.6%)  2,870 (11.4%) |
| **STDR**  No  Yes | 24,556 (98.6%)  340 (1.4%) | 24,883 (99.0%)  260 (1.0%) |

Abbreviations: T2DM- type 2 diabetes; eGFR-estimated glomerular filtration rate; HbA1c-glycated haemoglobin; HDL- high density lipoprotein; SBP-systolic blood pressure; BMI- Body mass index; antiHT- antihypertensive agents; AF-atrial fibrillation; HF-heart failure; MI-myocardial infarction; CHD-coronary artery disease; STDR-sight threatening diabetic retinopathy; SD-standard deviation; IQR-interquartile range

### **Table S5. 5-year incidence rates and time of stage 3 CKD**

| **Outcome measures** | | **London(N=20,510)** | **Wales(N=13,346)** |
| --- | --- | --- | --- |
| **Events** | | 1,378 | 656 |
| **Time** | **Total time at risk, years** | 75,420.8 | 49,352.5 |
|  | **Mean (SD)**  **Median (IQR) follow-up, years** | 3.7 (1.5)  4.4 (2.4-5) | 3.7 (1.5)  4.4 (2.5-5) |
| **Incidence rates by Age group, per 1,000 person-years (95% CI); number of events** | **Overall** | 18.3 (17.3-19.3);1,378 | 13.3 (12.3-14.3);656 |
|  | **<35** | 1.4 (0.7-2.9);7 | 0;0 |
|  | **36-40** | 3.3 (2.2-4.8);27 | 3.02(1.51,6.03);8 |
|  | **41-50** | 7.4 (6.3-8.6);159 | 4.04(2.96,5.50);40 |
|  | **51-60** | 16.8 (15.1-18.6);356 | 8.03(6.69,9.64);115 |
|  | **61-70** | 32.3 (29.3-35.5);410 | 18.52(16.38,20.94);255 |
|  | **71-80** | 60.0 (54.0-66.7);344 | 32.70(28.36,37.71);189 |
|  | **>80** | 85.4 (68.1-107.0);75 | 55.53(41.97,73.47);49 |

eGFR-estimated glomerular filtration rate; CKD-chronic kidney disease.

### **Table S6. Risk prediction equations for the 5-year risk of new onset stage 3+ CKD (reduced eGFR) for all models**

| **Model** | **Baseline survival estimate; re-calibrated to Wales** | | | | | **Linear predictor** |
| --- | --- | --- | --- | --- | --- | --- |
|  | **5-year; events= 1,378** | **4-year; events= 1,131** | **3-year; events= 818** | **2-year; events= 474** | **1-year; events= 120** |  |
| **Full model** | 0.9824; 0.9863 | 0.9872; 0.9900 | 0.9918; 0.9929 | 0.9958; 0.9962 | 0.9991; 0.9989 | 0.2074615*(if Black) +0.5816492*(ACR>=3 & ACR<30) + 1.212675 *(ACR>30) + 0.0510898*(log((duration+0.0027389526367188)/10) +1.075175776) + 0.15732*(has history of CVD) + 0.4256548*(on Insulin) + 0.0046519*(SBP-131.5080449) +0.2071413*( On antiHT) + 0.3863518*(has STDR) + 2.587995*(eGFR >60 & eGFR <70) + 1.500215*(eGFR>=70 & eGFR<=80) + 0.760282*(eGFR >80 & eGFR <90) + -0.2775169*(HDL -1.175928328) + -12.90798 *(((age/10)^-0.5)-0.4341229053) + 0.0034228*(HbA1c-65.75536883) + -0.126823*(If male) +9.378112*(On insulin)*(((age/10)^-0.5)-0.4341229053) |
| **Reduced model** | 0.9824; 0.9860 | 0.9872; 0.9898 | 0.9918; 0.9928 | 0.9958; 0.9962 | 0.9991; 0.9988 | 0.1981315*(if Black)+0.5873524*(ACR >=3 & ACR<=30) + 1.217188*(ACR>30) + 0.0525226*(log((duration +0.0027389526367188)/10) +1.075175776) + 0.4631486*(on Insulin) + 0.0045032*(SBP-131.5080449) +0.2317291*(On antiHT) + 2.595921*(eGFR >60 & eGFR<70) + 1.50606*(eGFR >= 70 & eGFR<=80) + 0.7668493*(eGFR >80 & eGFR <90) + -0.2726254*(HDL -1.175928328) + -13.12071*(((age/10)^-0.5)-0.4341229053) + 0.0034468*(HbA1c -65.76331125) + -0.1142733*(if male) + 9.211248*(On insulin)*(((age/10)^-0.5)-0.4341229053) |
| **Minimal-resources model** | 0.9823; 0.9864 | 0.9875; 0.9901 | 0.9920; 0.9930 | 0.9959; 0.9963 | 0.9991; 0.9989 | 0.1641753*(if Black) +0.6193761*(ACR>=3 & ACR<=30) + 1.26135*(ACR >30) + 0.0518075*((log((duration+0.0027389526367188)/10))+1.075175776) + 0.5047339*(On Insulin) + 0.0046369*(SBP-131.5080449) +0.210877*(On antiHT) + -12.42878*(((age/10)^-0.5)-0.4341229053) + -0.0593375*(if male) + 2.604593*(eGFR >60 & eGFR <70) +1.50991*(eGFR >=70 & eGFR<=80) + 0.7719732*(eGFR>80 & eGFR<90)+ 9.140567 *(On Insulin)*(((age/10)^-0.5)-0.4341229053) |

The equation is expressed as 1- s(t) = 1- s_0_(t)^exp(Xb)^ ,where s(t) is the probability of surviving and s_0_(t) is the baseline survival function at 5 years, X is the matrix which contains the values of the risk factors and b is a vector of the coefficients for each risk factor in the model (Xb is the linear predictor or prognostic index). Abbreviations: ACR, albumin: creatinine ratio; CVD, cardiovascular disease; antiHT, anti-hypertensive agents; STDR, sight-threatening diabetic retinopathy; eGFR, estimated glomerular filtration rate; HbA1c, haemoglobin A_1C_; duration, duration of type-2 diabetes; HDL- high density lipoprotein; SBP-systolic blood pressure; CKD-chronic kidney disease

### **Table S7. Sensitivity analysis 1: Multivariable cox regression model when including ACR measurements 24 months prior to and 6 months after baseline date**

| **Confirmed eGFR < 60 mL/min per 1.73m2 (at two time points), stage 3+ code or dialysis**  (N=26,239; Events=1,736; total time at risk, y=96,962.3) | | | | | | | | | |
| --- | --- | --- | --- | --- | --- | --- | --- | --- | --- |
| Patient Characteristic | **Full Model** | | | **Reduced model** | | | **Minimal – resources model** | | |
|  | **HR** | **P** | **95% CI** | **HR** | **P** | **95% CI** | **HR** | **P** | **95% CI** |
| Gender  Female  Male | Ref  0.88 | -  0.010 | -  0.80-0.97 | Ref  0.89 | -  0.019 | -  0.81-0.98 | Ref  0.94 | -  0.193 | -  0.85-1.03 |
| Ethnicity  Black† | 1.20 | 0.001 | 1.07-1.34 | 1.19 | 0.002 | 1.07-1.33 | 1.16 | 0.006 | 1.04-1.30 |
| eGFR , mL/min/1.73m2 (for age 54 years)  >=60 & <=70  >70 & <=80  > 80 & <90  >=90 | 13.14  4.31  2.16  Ref | <0.001  <0.001  <0.001  - | 10.95-15.76  3.57-5.21  1.75-2.66  - | 13.26  4.34  2.17 | <0.001  <0.001  <0.001 | 11.05-15.90  3.59-5.24  1.76-2.68 | 13.32  4.35  2.18  Ref | <0.001  <0.001  <0.001  - | 11.10-15.98  3.60-5.26  1.77-2.69  - |
| Albuminuria mg/mmol  No  Micro  Macro | Ref  1.76  3.23 | -  <0.001  <0.001 | -  1.58-1.96  2.67-3.91 | Ref  1.77  3.25 | -  <0.001  <0.001 | -  1.59-1.96  2.69-3.93 | Ref  1.83  3.41 | -  <0.001  <0.001 | -  1.65-2.03  2.83-4.12 |
| Systolic blood pressure, per 1 unit increase in mmHg | 1.005 | 0.001 | 1.002-1.008 | 1.005 | 0.001 | 1.002-1.007 | 1.004 | 0.001 | 1.002-1.008 |
| On Insulin (for age 54 years)  No  yes | Ref  1.43 | -  <0.001 | -  1.19-1.72 | Ref  1.48 | -  <0.001 | -  1.23-1.79 | Ref  1.56 | -  <0.001 | -  1.30-1.87 |
| On AntiHT Agents ±  No  Yes | Ref  1.29 | -  <0.001 | -  1.15-1.44 | Ref  1.32 | -  <0.001 | -  1.18-1.47 | Ref  1.29 | -  <0.001 | -  1.15-1.43 |
| HbA1c, per 1 unit increase in mmol/L | 1.004 | <0.001 | 1.002-1.007 | 1.004 | <0.001 | 1.002-1.007 | - | - | - |
| HDL, per 1 unit increase in mmol/L | 0.76 | 0.001 | 0.65-0.89 | 0.77 | 0.001 | 0.66-0.90 | - | - | - |
| Presence of STDR  No  Yes | Ref  1.51 | -  0.002 | -  1.16-1.98 | - | - | - | - | - | - |
| CVD history ¥  No  Yes | Ref  1.14 | -  0.035 | -  1.01-1.29 | - | - | - | - | - | - |
| C-statistic(95% CI): Development | 0.853(0.845-0.861) | | | 0.852(0.845-0.861) | | | 0.852(0.844-0.860) | | |

Abbreviations: T2DM, type-2 diabetes mellites ;eGFR estimated glomerular filtration rate; AntiHT, Anti-hypertensive; CVD, cardiovascular disease; HbA1c, haemoglobin A_1c_; STDR sight-threatening diabetic retinopathy

Models included fractional polynomial terms for age and duration as follows:

Age term was (age/10)^-0.5 -0.43

Duration term was ln(duration+0.003)/10 +1.10

Models included a two-way interaction term between age and insulin use

¥ CVD includes MI, stroke, AF, HF and CHD

± Anti-hypertensive agents include ARB /ACE drugs

†Compared to patients in the White, South Asian, Mixed or Other groups

### **Table S8. Sensitivity analysis 2: C-statistics(SE) and calibration slopes(SE) comparison in subgroups (in Wales)**

|  |  |  |  |  | **C-statistic** | | | **Calibration slope** | | |
| --- | --- | --- | --- | --- | --- | --- | --- | --- | --- | --- |
|  |  |  | **N** | **Events** | **Full model** | **Reduced model** | **Minimal-resources model** | **Full model** | **Reduced model** | **Minimal-resources model** |
| **Non-modifiable risk factors** | **Duration** | **Newly diagnosed diabetes (duration 0 years)** | 11474 | 518 | 0.826(0.008) | 0.826(0.008) | 0.823(0.009) | 1.034(0.04) | 1.039(0.04) | 1.024(0.04) |
|  |  | **>0 years** | 1872 | 138 | 0.812(0.017) | 0.811(0.017) | 0.811(0.017) | 1.028(0.079) | 1.033(0.08) | 1.035(0.081) |
|  | **Age** | **<60 years** | 7491 | 153 | 0.815(0.016) | 0.814(0.016) | 0.815(0.016) | 1.01(0.067) | 1.01(0.068) | 1.02(0.069) |
|  |  | **>=60 years** | 5855 | 503 | 0.77(0.01) | 0.77(0.01) | 0.765(0.01) | 0.982(0.047) | 0.992(0.047) | 0.973(0.047) |
|  | **Gender** | **Male** | 8591 | 369 | 0.829(0.01) | 0.829(0.01) | 0.827(0.01) | 1.035(0.046) | 1.043(0.047) | 1.032(0.047) |
|  |  | **Female** | 4755 | 287 | 0.819(0.012) | 0.818(0.012) | 0.815(0.012) | 0.995(0.053) | 1.00(0.053) | 0.992(0.053) |

Abbreviations: ACR-albumin creatinine ratio; DR-diabetic retinopathy. C-Statistics and calibration slopes in validation cohort generated using R (Version 4.0.2)

### **Table S9. Risk score points**

| **Age, years** | | | **SBP, mmHg** | | **Duration, years** | | **eGFR, ml/min/1.73m^2^** | | **ACR, mg/mmol** | | **Ethnicity** | | **AntiHT** | | **Gender** | |
| --- | --- | --- | --- | --- | --- | --- | --- | --- | --- | --- | --- | --- | --- | --- | --- | --- |
| **Age** | **Points** | | **SBP** | **Points** | **Duration** | **Points** | **eGFR** | **Points** | **ACR** | **Points** | **Ethnicity** | **Points** | **AntiHT** | **Points** | **Gender** | **Points** |
|  |  |  |  |  |  |  |  |  |  |  |  |  |  |  |  |  |
|  |  |  |  |  |  |  |  |  |  |  |  |  |  |  |  |  |
|  |  |  |  |  |  |  |  |  |  |  |  |  |  |  |  |  |
|  | **No**  **Insulin** | **Insulin** |  |  |  |  |  |  |  |  |  |  |  |  |  |  |
| 19 | 0 | 64 | 70-75 | 0 | 0 | 0 | 60-70 | 52 | <3 | 0 | Other | 0 | No | 0 | Female | 1 |
| 20 | 5 | 65 | 76-86 | 1 | 1 | 6 | 71-80 | 30 | 3-30 | 12 | Black | 3 | Yes | 4 | Male | 0 |
| 21 | 9 | 66 | 87-96 | 2 | 2-3 | 7 | 81-89 | 15 | >30 | 25 |  |  |  |  |  |  |
| 22 | 13 | 67 | 97-107 | 3 | 4-9 | 8 | 90+ | 0 |  |  |  |  |  |  |  |  |
| 23 | 16 | 68 | 108-118 | 4 | 10-25 | 9 |  |  |  |  |  |  |  |  |  |  |
| 24 | 20 | 69 | 119-129 | 5 | 26+ | 10 |  |  |  |  |  |  |  |  |  |  |
| 25 | 23 | 70 | 130-139 | 6 |  |  |  |  |  |  |  |  |  |  |  |  |
| 26 | 26 | 71 | 140-150 | 7 |  |  |  |  |  |  |  |  |  |  |  |  |
| 27 | 29 | 71 | 151-161 | 8 |  |  |  |  |  |  |  |  |  |  |  |  |
| 28 | 32 | 72 | 162-172 | 9 |  |  |  |  |  |  |  |  |  |  |  |  |
| 29 | 34 | 73 | 173-182 | 10 |  |  |  |  |  |  |  |  |  |  |  |  |
| 30 | 37 | 73 | 183-193 | 11 |  |  |  |  |  |  |  |  |  |  |  |  |
| 31 | 39 | 74 | 194-204 | 12 |  |  |  |  |  |  |  |  |  |  |  |  |
| 32 | 42 | 75 | 205-215 | 13 |  |  |  |  |  |  |  |  |  |  |  |  |
| 33 | 44 | 75 | 216-225 | 14 |  |  |  |  |  |  |  |  |  |  |  |  |
| 34 | 46 | 76 | 226-228 | 15 |  |  |  |  |  |  |  |  |  |  |  |  |
| 35 | 48 | 76 |  |  |  |  |  |  |  |  |  |  |  |  |  |  |
| 36 | 49 | 77 |  |  |  |  |  |  |  |  |  |  |  |  |  |  |
| 37 | 51 | 77 |  |  |  |  |  |  |  |  |  |  |  |  |  |  |
| 38 | 53 | 78 |  |  |  |  |  |  |  |  |  |  |  |  |  |  |
| 39 | 55 | 78 |  |  |  |  |  |  |  |  |  |  |  |  |  |  |
| 40 | 56 | 78 |  |  |  |  |  |  |  |  |  |  |  |  |  |  |
| 41 | 58 | 79 |  |  |  |  |  |  |  |  |  |  |  |  |  |  |
| 42 | 59 | 79 |  |  |  |  |  |  |  |  |  |  |  |  |  |  |
| 43 | 61 | 80 |  |  |  |  |  |  |  |  |  |  |  |  |  |  |
| 44 | 62 | 80 |  |  |  |  |  |  |  |  |  |  |  |  |  |  |
| 45 | 63 | 80 |  |  |  |  |  |  |  |  |  |  |  |  |  |  |
| 46 | 65 | 81 |  |  |  |  |  |  |  |  |  |  |  |  |  |  |
| 47 | 66 | 81 |  |  |  |  |  |  |  |  |  |  |  |  |  |  |
| 48 | 67 | 81 |  |  |  |  |  |  |  |  |  |  |  |  |  |  |
| 49 | 68 | 82 |  |  |  |  |  |  |  |  |  |  |  |  |  |  |
| 50 | 69 | 82 |  |  |  |  |  |  |  |  |  |  |  |  |  |  |
| 51 | 70 | 82 |  |  |  |  |  |  |  |  |  |  |  |  |  |  |
| 52 | 72 | 82 |  |  |  |  |  |  |  |  |  |  |  |  |  |  |
| 53 | 73 | 83 |  |  |  |  |  |  |  |  |  |  |  |  |  |  |
| 54 | 74 | 83 |  |  |  |  |  |  |  |  |  |  |  |  |  |  |
| 55 | 75 | 83 |  |  |  |  |  |  |  |  |  |  |  |  |  |  |
| 56 | 76 | 84 |  |  |  |  |  |  |  |  |  |  |  |  |  |  |
| 57 | 76 | 84 |  |  |  |  |  |  |  |  |  |  |  |  |  |  |
| 58 | 77 | 84 |  |  |  |  |  |  |  |  |  |  |  |  |  |  |
| 59 | 78 | 84 |  |  |  |  |  |  |  |  |  |  |  |  |  |  |
| 60 | 79 | 84 |  |  |  |  |  |  |  |  |  |  |  |  |  |  |
| 61 | 80 | 85 |  |  |  |  |  |  |  |  |  |  |  |  |  |  |
| 62 | 81 | 85 |  |  |  |  |  |  |  |  |  |  |  |  |  |  |
| 63 | 82 | 85 |  |  |  |  |  |  |  |  |  |  |  |  |  |  |
| 64 | 82 | 85 |  |  |  |  |  |  |  |  |  |  |  |  |  |  |
| 65 | 83 | 86 |  |  |  |  |  |  |  |  |  |  |  |  |  |  |
| 66 | 84 | 86 |  |  |  |  |  |  |  |  |  |  |  |  |  |  |
| 67 | 85 | 86 |  |  |  |  |  |  |  |  |  |  |  |  |  |  |
| 68 | 85 | 86 |  |  |  |  |  |  |  |  |  |  |  |  |  |  |
| 69 | 86 | 86 |  |  |  |  |  |  |  |  |  |  |  |  |  |  |
| 70 | 87 | 86 |  |  |  |  |  |  |  |  |  |  |  |  |  |  |
| 71 | 87 | 87 |  |  |  |  |  |  |  |  |  |  |  |  |  |  |
| 72 | 88 | 87 |  |  |  |  |  |  |  |  |  |  |  |  |  |  |
| 73 | 89 | 87 |  |  |  |  |  |  |  |  |  |  |  |  |  |  |
| 74 | 89 | 87 |  |  |  |  |  |  |  |  |  |  |  |  |  |  |
| 75 | 90 | 87 |  |  |  |  |  |  |  |  |  |  |  |  |  |  |
| 76 | 90 | 87 |  |  |  |  |  |  |  |  |  |  |  |  |  |  |
| 77 | 91 | 88 |  |  |  |  |  |  |  |  |  |  |  |  |  |  |
| 78 | 92 | 88 |  |  |  |  |  |  |  |  |  |  |  |  |  |  |
| 79 | 92 | 88 |  |  |  |  |  |  |  |  |  |  |  |  |  |  |
| 80 | 93 | 88 |  |  |  |  |  |  |  |  |  |  |  |  |  |  |
| 81 | 93 | 88 |  |  |  |  |  |  |  |  |  |  |  |  |  |  |
| 82 | 94 | 88 |  |  |  |  |  |  |  |  |  |  |  |  |  |  |
| 83 | 94 | 89 |  |  |  |  |  |  |  |  |  |  |  |  |  |  |
| 84 | 95 | 89 |  |  |  |  |  |  |  |  |  |  |  |  |  |  |
| 85 | 95 | 89 |  |  |  |  |  |  |  |  |  |  |  |  |  |  |
| 86 | 96 | 89 |  |  |  |  |  |  |  |  |  |  |  |  |  |  |
| 87 | 96 | 89 |  |  |  |  |  |  |  |  |  |  |  |  |  |  |
| 88 | 97 | 89 |  |  |  |  |  |  |  |  |  |  |  |  |  |  |
| 89 | 97 | 89 |  |  |  |  |  |  |  |  |  |  |  |  |  |  |
| 90 | 98 | 89 |  |  |  |  |  |  |  |  |  |  |  |  |  |  |
| 91 | 98 | 90 |  |  |  |  |  |  |  |  |  |  |  |  |  |  |
| 92 | 99 | 90 |  |  |  |  |  |  |  |  |  |  |  |  |  |  |
| 93 | 99 | 90 |  |  |  |  |  |  |  |  |  |  |  |  |  |  |
| 94 | 100 | 90 |  |  |  |  |  |  |  |  |  |  |  |  |  |  |
| 95 | 100 | 90 |  |  |  |  |  |  |  |  |  |  |  |  |  |  |

Abbreviations: SBP, systolic blood pressure; eGFR, estimated glomerular filtration rate; ACR, albumin: creatinine ratio; AntiHT, anti-hypertensive use.

Risk score points were generated from the regression nomogram using rms library (39) in R . Points for continuous variables were generated per unit increase of the variable in question. Total points can be converted into predicted probabilities(risks) using the following formula = 1-0.9828^exp(-4.34+0.05*Total points) for risks in London and 0.9864^exp(-4.34+0.05*Total points) for re-calibrated risks (Wales). Alternatively, predicted probabilities can be ascertained directly from the total points given in table S9. For instance, for an individual with 5 years duration, age 60, taking antihypertensive medication, on insulin, female, black race, microalbuminuria, eGFR 75 and blood pressure 140, the points system gives a 5-year estimate of risk as: $Total points=84+7+8+30+12+3+4+1=149\Rightarrow LP=-4.34+0.05*Total points=2.961\Rightarrow event risk \%= 1-{0.9828}^{exp\left( 2.961 \right)}=28.5\%$. The cox model estimates risk as: $LP = 3.118 \Rightarrow event risk \%=1-{0.9828}^{exp\left( 3.118 \right)}=32.4\% .$

### **Table S10. Total points to predicted probabilities**

| **Total points** | **5-year Risk** | **5-year recalibrated risk** |
| --- | --- | --- |
| 0 | 0.0% | 0.0% |
| 1 | 0.0% | 0.0% |
| 2 | 0.0% | 0.0% |
| 3 | 0.0% | 0.0% |
| 4 | 0.0% | 0.0% |
| 5 | 0.0% | 0.0% |
| 6 | 0.0% | 0.0% |
| 7 | 0.0% | 0.0% |
| 8 | 0.0% | 0.0% |
| 9 | 0.0% | 0.0% |
| 10 | 0.0% | 0.0% |
| 11 | 0.0% | 0.0% |
| 12 | 0.0% | 0.0% |
| 13 | 0.0% | 0.0% |
| 14 | 0.0% | 0.0% |
| 15 | 0.0% | 0.0% |
| 16 | 0.1% | 0.0% |
| 17 | 0.1% | 0.0% |
| 18 | 0.1% | 0.0% |
| 19 | 0.1% | 0.0% |
| 20 | 0.1% | 0.0% |
| 21 | 0.1% | 0.1% |
| 22 | 0.1% | 0.1% |
| 23 | 0.1% | 0.1% |
| 24 | 0.1% | 0.1% |
| 25 | 0.1% | 0.1% |
| 26 | 0.1% | 0.1% |
| 27 | 0.1% | 0.1% |
| 28 | 0.1% | 0.1% |
| 29 | 0.1% | 0.1% |
| 30 | 0.1% | 0.1% |
| 31 | 0.1% | 0.1% |
| 32 | 0.1% | 0.1% |
| 33 | 0.1% | 0.1% |
| 34 | 0.1% | 0.1% |
| 35 | 0.1% | 0.1% |
| 36 | 0.1% | 0.1% |
| 37 | 0.1% | 0.1% |
| 38 | 0.2% | 0.1% |
| 39 | 0.2% | 0.1% |
| 40 | 0.2% | 0.1% |
| 41 | 0.2% | 0.1% |
| 42 | 0.2% | 0.1% |
| 43 | 0.2% | 0.2% |
| 44 | 0.2% | 0.2% |
| 45 | 0.2% | 0.2% |
| 46 | 0.2% | 0.2% |
| 47 | 0.2% | 0.2% |
| 48 | 0.2% | 0.2% |
| 49 | 0.3% | 0.2% |
| 50 | 0.3% | 0.2% |
| 51 | 0.3% | 0.2% |
| 52 | 0.3% | 0.2% |
| 53 | 0.3% | 0.3% |
| 54 | 0.3% | 0.3% |
| 55 | 0.4% | 0.3% |
| 56 | 0.4% | 0.3% |
| 57 | 0.4% | 0.3% |
| 58 | 0.4% | 0.3% |
| 59 | 0.4% | 0.3% |
| 60 | 0.5% | 0.4% |
| 61 | 0.5% | 0.4% |
| 62 | 0.5% | 0.4% |
| 63 | 0.5% | 0.4% |
| 64 | 0.6% | 0.4% |
| 65 | 0.6% | 0.5% |
| 66 | 0.6% | 0.5% |
| 67 | 0.6% | 0.5% |
| 68 | 0.7% | 0.5% |
| 69 | 0.7% | 0.6% |
| 70 | 0.7% | 0.6% |
| 71 | 0.8% | 0.6% |
| 72 | 0.8% | 0.7% |
| 73 | 0.9% | 0.7% |
| 74 | 0.9% | 0.7% |
| 75 | 1.0% | 0.8% |
| 76 | 1.0% | 0.8% |
| 77 | 1.1% | 0.8% |
| 78 | 1.1% | 0.9% |
| 79 | 1.2% | 0.9% |
| 80 | 1.2% | 1.0% |
| 81 | 1.3% | 1.0% |
| 82 | 1.4% | 1.1% |
| 83 | 1.4% | 1.1% |
| 84 | 1.5% | 1.2% |
| 85 | 1.6% | 1.2% |
| 86 | 1.7% | 1.3% |
| 87 | 1.7% | 1.4% |
| 88 | 1.8% | 1.4% |
| 89 | 1.9% | 1.5% |
| 90 | 2.0% | 1.6% |
| 91 | 2.1% | 1.7% |
| 92 | 2.2% | 1.8% |
| 93 | 2.3% | 1.8% |
| 94 | 2.5% | 1.9% |
| 95 | 2.6% | 2.0% |
| 96 | 2.7% | 2.1% |
| 97 | 2.8% | 2.3% |
| 98 | 3.0% | 2.4% |
| 99 | 3.1% | 2.5% |
| 100 | 3.3% | 2.6% |
| 101 | 3.5% | 2.7% |
| 102 | 3.6% | 2.9% |
| 103 | 3.8% | 3.0% |
| 104 | 4.0% | 3.2% |
| 105 | 4.2% | 3.3% |
| 106 | 4.4% | 3.5% |
| 107 | 4.7% | 3.7% |
| 108 | 4.9% | 3.9% |
| 109 | 5.1% | 4.1% |
| 110 | 5.4% | 4.3% |
| 111 | 5.7% | 4.5% |
| 112 | 5.9% | 4.7% |
| 113 | 6.2% | 4.9% |
| 114 | 6.5% | 5.2% |
| 115 | 6.9% | 5.5% |
| 116 | 7.2% | 5.7% |
| 117 | 7.6% | 6.0% |
| 118 | 7.9% | 6.3% |
| 119 | 8.3% | 6.6% |
| 120 | 8.7% | 6.9% |
| 121 | 9.1% | 7.3% |
| 122 | 9.6% | 7.7% |
| 123 | 10.1% | 8.0% |
| 124 | 10.5% | 8.4% |
| 125 | 11.1% | 8.8% |
| 126 | 11.6% | 9.3% |
| 127 | 12.1% | 9.7% |
| 128 | 12.7% | 10.2% |
| 129 | 13.3% | 10.7% |
| 130 | 14.0% | 11.2% |
| 131 | 14.6% | 11.7% |
| 132 | 15.3% | 12.3% |
| 133 | 16.0% | 12.9% |
| 134 | 16.8% | 13.5% |
| 135 | 17.6% | 14.1% |
| 136 | 18.4% | 14.8% |
| 137 | 19.2% | 15.5% |
| 138 | 20.1% | 16.2% |
| 139 | 21.0% | 17.0% |
| 140 | 22.0% | 17.8% |
| 141 | 23.0% | 18.6% |
| 142 | 24.0% | 19.5% |
| 143 | 25.0% | 20.3% |
| 144 | 26.1% | 21.3% |
| 145 | 27.3% | 22.2% |
| 146 | 28.5% | 23.2% |
| 147 | 29.7% | 24.3% |
| 148 | 30.9% | 25.3% |
| 149 | 32.2% | 26.4% |
| 150 | 33.6% | 27.6% |
| 151 | 34.9% | 28.8% |
| 152 | 36.4% | 30.0% |
| 153 | 37.8% | 31.3% |
| 154 | 39.3% | 32.6% |
| 155 | 40.8% | 33.9% |
| 156 | 42.4% | 35.3% |
| 157 | 44.0% | 36.7% |
| 158 | 45.7% | 38.2% |
| 159 | 47.3% | 39.7% |
| 160 | 49.0% | 41.3% |
| 161 | 50.8% | 42.8% |
| 162 | 52.5% | 44.5% |
| 163 | 54.3% | 46.1% |
| 164 | 56.1% | 47.8% |
| 165 | 57.9% | 49.5% |
| 166 | 59.8% | 51.2% |
| 167 | 61.6% | 53.0% |
| 168 | 63.4% | 54.8% |
| 169 | 65.3% | 56.6% |
| 170 | 67.1% | 58.4% |
| 171 | 68.9% | 60.2% |
| 172 | 70.7% | 62.1% |
| 173 | 72.5% | 63.9% |
| 174 | 74.3% | 65.8% |
| 175 | 76.0% | 67.6% |
| 176 | 77.7% | 69.4% |
| 177 | 79.4% | 71.2% |
| 178 | 81.0% | 73.0% |
| 179 | 82.5% | 74.7% |
| 180 | 84.0% | 76.5% |
| 181 | 85.4% | 78.1% |
| 182 | 86.8% | 79.8% |
| 183 | 88.1% | 81.4% |
| 184 | 89.3% | 82.9% |
| 185 | 90.5% | 84.4% |
| >185 | >90.5% | >84.4% |

Total points to predicted risks in London and recalibrated risks in Wales

### **Table S11. Agreement between risk estimates based on points system and risk estimates based on multivariable cox model**

|  | **Linear predictor based on points system** | | | | |  |
| --- | --- | --- | --- | --- | --- | --- |
| **Linear predictor estimate based on mfp model** |  | **≤-0.69** | **>-0.69&≤0.72** | **>0.72&≤2.36** | **>2.36** | **Total** |
|  | **≤-0.69** | 3269 | 7 | 0 | 0 | 3276 |
|  | **>-0.69 & ≤0.72** | 137 | 6828 | 0 | 0 | 6965 |
|  | **>0.72&≤2.36** | 0 | 463 | 6511 | 0 | 6974 |
|  | **>2.36** | 0 | 0 | 277 | 3018 | 3295 |
| **Total** |  | 3406 | 7298 | 6788 | 3018 | 20510 |

Abbreviations: mfp; multivariable fractional polynomial; ASE, approximate standard error

Linear predictor categorised by cox’s cut-points (16^th^, 50^th^ and 84^th^ percentiles)

The weighted kappa statistic computes the corrected and standardised weighted diagonal sum of the relative frequencies.

Linear weights are defined by $1-\left( \frac{abs\left( i-j \right)}{r-1} \right)$or quadratic (Fleiss-Cohen) by $1 -(abs(i - j)^2 / (r - 1)^2)$, where i,j are

row, column index respectively and r, the number of categories

Linear (equal-spacing) Kappa (κ) = 0.9583 (ASE=0.001389)

Quadratic (Fleiss-Cohen) Kappa (κ) = 0.9756 (ASE=0.008269)

### **Supplement Bibliography**

[1] Nelson RG, Grams ME, Ballew SH, et al. (2019) Development of Risk Prediction Equations for Incident Chronic Kidney Disease. Jama 322(21): 2104-2114. 10.1001/jama.2019.17379 %J JAMA

[2] Basu S, Sussman JB, Berkowitz SA, Hayward RA, Yudkin JS (2017) Development and validation of Risk Equations for Complications Of type 2 Diabetes (RECODe) using individual participant data from randomised trials. Lancet Diabetes Endocrinol 5(10): 788-798. 10.1016/s2213-8587(17)30221-8

[3] Mise K, Hoshino J, Ueno T, et al. (2016) Prognostic Value of Tubulointerstitial Lesions, Urinary N-Acetyl-β-d-Glucosaminidase, and Urinary β2-Microglobulin in Patients with Type 2 Diabetes and Biopsy-Proven Diabetic Nephropathy. Clinical journal of the American Society of Nephrology : CJASN 11(4): 593-601. 10.2215/cjn.04980515

[4] Mocroft A, Lundgren JD, Ross M, et al. (2015) Development and validation of a risk score for chronic kidney disease in HIV infection using prospective cohort data from the D:A:D study. PLoS medicine 12(3): e1001809-e1001809. 10.1371/journal.pmed.1001809

[5] O'Seaghdha CM, Lyass A, Massaro JM, et al. (2012) A risk score for chronic kidney disease in the general population. The American journal of medicine 125(3): 270-277. 10.1016/j.amjmed.2011.09.009

[6] Alssema M, Newson RS, Bakker SJL, et al. (2012) One risk assessment tool for cardiovascular disease, type 2 diabetes, and chronic kidney disease. Diabetes care 35(4): 741-748. 10.2337/dc11-1417

[7] Jardine MJ, Hata J, Woodward M, et al. (2012) Prediction of kidney-related outcomes in patients with type 2 diabetes. American journal of kidney diseases : the official journal of the National Kidney Foundation 60(5): 770-778. 10.1053/j.ajkd.2012.04.025

[8] O'Seaghdha CM, Yang Q, Wu H, Hwang S-J, Fox CS (2012) Performance of a genetic risk score for CKD stage 3 in the general population. Am J Kidney Dis 59(1): 19-24. 10.1053/j.ajkd.2011.08.030

[9] Ando M, Yanagisawa N, Ajisawa A, Tsuchiya K, Nitta K (2011) A simple model for predicting incidence of chronic kidney disease in HIV-infected patients. Clinical and Experimental Nephrology 15(2): 242-247. 10.1007/s10157-010-0393-x

[10] Fox CS, Gona P, Larson MG, et al. (2010) A multi-marker approach to predict incident CKD and microalbuminuria. J Am Soc Nephrol 21(12): 2143-2149. 10.1681/ASN.2010010085

[11] Hippisley-Cox J, Coupland C (2010) Predicting the risk of Chronic Kidney Disease in Men and Women in England and Wales: prospective derivation and external validation of the QKidneyScores. BMC Family Practice 11(1): 49. 10.1186/1471-2296-11-49

[12] Chien KL, Lin HJ, Lee BC, Hsu HC, Lee YT, Chen MF (2010) A prediction model for the risk of incident chronic kidney disease. The American journal of medicine 123(9): 836-846.e832. 10.1016/j.amjmed.2010.05.010

[13] Kshirsagar AV, Bang H, Bomback AS, et al. (2008) A simple algorithm to predict incident kidney disease. Archives of internal medicine 168(22): 2466-2473. 10.1001/archinte.168.22.2466
